# Internet-accessed sexually transmitted infection (e-STI) testing and results service: A randomised, single-blind, controlled trial

**DOI:** 10.1371/journal.pmed.1002479

**Published:** 2017-12-27

**Authors:** Emma Wilson, Caroline Free, Tim P. Morris, Jonathan Syred, Irrfan Ahamed, Anatole S. Menon-Johansson, Melissa J. Palmer, Sharmani Barnard, Emma Rezel, Paula Baraitser

**Affiliations:** 1 Faculty of Epidemiology and Population Health, London School of Hygiene & Tropical Medicine, London, United Kingdom; 2 Medical Research Council Clinical Trials Unit at UCL, London, United Kingdom; 3 King’s Centre for Global Health and Health Partnerships, School of Population Health & Environmental Sciences, King’s College London, London, United Kingdom; 4 Burrell Street Sexual Health Clinic, Guy’s and St Thomas’ NHS Foundation Trust, London, United Kingdom; 5 Department of Sexual Health and HIV, King’s College Hospital NHS Foundation Trust, London, United Kingdom; World Health Organization, SWITZERLAND

## Abstract

**Background:**

Internet-accessed sexually transmitted infection testing (e-STI testing) is increasingly available as an alternative to testing in clinics. Typically this testing modality enables users to order a test kit from a virtual service (via a website or app), collect their own samples, return test samples to a laboratory, and be notified of their results by short message service (SMS) or telephone. e-STI testing is assumed to increase access to testing in comparison with face-to-face services, but the evidence is unclear. We conducted a randomised controlled trial to assess the effectiveness of an e-STI testing and results service (chlamydia, gonorrhoea, HIV, and syphilis) on STI testing uptake and STI cases diagnosed.

**Methods and findings:**

The study took place in the London boroughs of Lambeth and Southwark. Between 24 November 2014 and 31 August 2015, we recruited 2,072 participants, aged 16–30 years, who were resident in these boroughs, had at least 1 sexual partner in the last 12 months, stated willingness to take an STI test, and had access to the internet. Those unable to provide consent and unable to read English were excluded. Participants were randomly allocated to receive 1 text message with the web link of an e-STI testing and results service (intervention group) or to receive 1 text message with the web link of a bespoke website listing the locations, contact details, and websites of 7 local sexual health clinics (control group). Participants were free to use any other services or interventions during the study period. The primary outcomes were self-reported STI testing at 6 weeks, verified by patient record checks, and self-reported STI diagnosis at 6 weeks, verified by patient record checks. Secondary outcomes were the proportion of participants prescribed treatment for an STI, time from randomisation to completion of an STI test, and time from randomisation to treatment of an STI. Participants were sent a £10 cash incentive on submission of self-reported data. We completed all follow-up, including patient record checks, by 17 June 2016. Uptake of STI testing was increased in the intervention group at 6 weeks (50.0% versus 26.6%, relative risk [RR] 1.87, 95% CI 1.63 to 2.15, *P <* 0.001). The proportion of participants diagnosed was 2.8% in the intervention group versus 1.4% in the control group (RR 2.10, 95% CI 0.94 to 4.70, *P =* 0.079). No evidence of heterogeneity was observed for any of the pre-specified subgroup analyses. The proportion of participants treated was 1.1% in the intervention group versus 0.7% in the control group (RR 1.72, 95% CI 0.71 to 4.16, *P =* 0.231). Time to test, was shorter in the intervention group compared to the control group (28.8 days versus 36.5 days, *P <* 0.001, test for difference in restricted mean survival time [RMST]), but no differences were observed for time to treatment (83.2 days versus 83.5 days, *P =* 0.51, test for difference in RMST). We were unable to recruit the planned 3,000 participants and therefore lacked power for the analyses of STI diagnoses and STI cases treated.

**Conclusions:**

The e-STI testing service increased uptake of STI testing for all groups including high-risk groups. The intervention required people to attend clinic for treatment and did not reduce time to treatment. Service innovations to improve treatment rates for those diagnosed online are required and could include e-treatment and postal treatment services. e-STI testing services require long-term monitoring and evaluation.

**Trial registration:**

ISRCTN Registry ISRCTN13354298.

## Abstract 2

In a single-blind randomised trial, Emma Wilson and colleagues assess the impact of an e-STI service on testing uptake and cases diagnosed.

## Author summary

**Why was this study done?:**

- Internet-accessed sexually transmitted infection testing (e-STI testing) is increasingly available. Yet the effectiveness of e-STI testing compared to face-to-face services is unclear.

**What did the researchers do and find?:**

- We conducted a large randomised controlled trial in London, United Kingdom, to assess the effects of an e-STI testing and results service (SH:24) on uptake of STI testing and STI cases diagnosed and treated, when delivered alongside usual care.
- The intervention offered postal self-sampling test kits for chlamydia, gonorrhoea, HIV, and syphilis, results delivered via text message or telephone, and web-based safer sex health information.
- We found that e-STI testing almost doubled uptake of testing among all groups, including groups at high risk for STIs.
- A larger study is required to understand the effects of e-STI testing on STI diagnosis and STI cases treated.
- Further research is required to evaluate the proportion of those diagnosed who are treated. Our findings suggest that additional inputs may be required to strengthen linkages to treatment (e.g., postal treatment).

**What do these findings mean?:**

- e-STI testing should be considered as a complement to existing services and as an effective measure to increase uptake of STI testing.
- The effect of e-STI testing on cases diagnosed and treated requires further evaluation.

## Introduction

Sexually transmitted infections (STIs) continue to be a global public health concern, with an estimated 357 million new infections of curable STIs (chlamydia, gonorrhoea, syphilis, and trichomoniasis) each year [1]. In England, there were 436,928 new diagnoses of STIs and 5,684 new cases of HIV in 2015 [2,3]. The burden of infection is disproportionately high among young adults (under 25 years), men who have sex with men (MSM), and black and minority ethnic (BME) groups [4].

Left undiagnosed and untreated, curable STIs such as chlamydia, trichomoniasis, gonorrhoea, and syphilis can facilitate the transmission of HIV and can cause sub-fertility, ectopic pregnancy, chronic pelvic pain, neurological and cardiovascular disease, neonatal mortality, and infant morbidities [5]. Undiagnosed HIV and late diagnosis of HIV lead to diminished health outcomes and reduced life expectancy [6].

Increasing testing, diagnosis, and treatment of STIs and reducing time to treatment is a global priority to reduce the prevalence of STIs and their associated sequelae [7,8]. In the UK, STI testing coverage remains sub-optimal. The 3rd National Survey of Sexual Attitudes and Lifestyles (NATSAL) found that two-thirds of 16–44-year-olds who tested positive for chlamydia had not had a chlamydia test in the past 12 months [9]. Further, timely diagnosis of HIV is a challenge [10]. In 2015, 39% of adults diagnosed with HIV in the UK were diagnosed late (CD4 count < 350 cells/mm^3^) [3]. Interventions that increase access among high-risk and hard-to-reach groups are needed to maximise the public health benefits of STI testing.

Digital technologies are increasingly utilised to deliver sexual health interventions (e-sexual health) [11,12]. These include internet-accessed STI testing (e-STI testing). Typically this testing modality enables users to order a test kit from a virtual service (via a website or app), collect their own samples, return test samples to a laboratory, and be notified of their results by short message service (SMS) text message or telephone [13,14].

e-STI testing may bypass the inconvenience and stigma associated with face-to-face services [15,16], and overcome supply constraints where clinical services are scarce [11,17]. In doing so, it could expand access to populations who do not use face-to-face services [18,19]. Shifting tasks to patients via virtual services, particularly for non-complex testing and treatment, may prove cost-effective [20].

Public sector providers in the UK, Canada, the United States, Australia, and some European countries offer e-STI testing to high-risk groups [21–26]. Yet the international evidence base on e-STI testing is scant. To our knowledge there have been no randomised controlled trials evaluating the effect of internet-based services offering testing for chlamydia, gonorrhoea, syphilis, and HIV on testing, diagnosis, or treatment of STIs. In this trial we assessed the effects of an e-STI testing and results service (SH:24) on uptake of STI testing and STI cases diagnosed and treated, when delivered alongside usual care. The version of SH:24 evaluated offered postal self-sampling test kits for chlamydia, gonorrhoea, HIV, and syphilis; results delivered via text message or telephone; and web-based safer sex health information.

## Methods

We carried out a single-blind randomised controlled trial of an e-STI testing and results service. The trial was conducted in London, UK, and participants were recruited between 24 November 2014 and 31 August 2015. Ethical approval was obtained from the National Research Ethics Service (NRES) Committee London–Camberwell St Giles (Ref 14/LO/1477). The trial protocol was accepted for publication in April 2015 and was published in January 2016 [27,28].

Young people aged 16 to 30 years of age, resident in the London boroughs of Lambeth and Southwark, sexually active (at least 1 sexual partner in the last 12 months), with stated willingness to take an STI test, and with access to the internet were eligible for inclusion. People who were unable to read English (the websites were only in English) or unable to provide consent were excluded.

We recruited in community settings to reach individuals who may not use conventional STI testing services. We utilised both face-to-face and online recruitment strategies. We promoted the trial in universities, further education colleges, market stalls, barber shops, bars, and nightclubs in South East London and via Facebook, Twitter, and Grindr (a dating application for gay and bisexual men). Advocacy and health promotion groups advertised the trial among their networks. The study was promoted in conjunction with a health promotion message, to motivate participants to join the trial and consider taking an STI test.

Research assistants assessed eligibility, provided study information, obtained written consent, and collected baseline data. Alternatively, participants read the information, entered their eligibility data, provided online consent, and entered their baseline data on the trial website. An independent computer-based randomisation programme allocated participants to the intervention or control group. Participants were sent 1 automated SMS text message with the uniform resource locator (URL) of the intervention or control STI services according to their allocation.

The randomisation system utilised a minimisation algorithm balancing for gender (male, female, transgender), age (16–19, 20–24, 25–30 years), number of sexual partners in last 12 months (1, 2+), and sexual orientation (MSM, all other groups). All factors had equal weight in determining marginal imbalance. To minimise imbalances on these selected factors, allocation was weighted towards the underrepresented group using a probability of 0.8. In the case of equal representation, participants were allocated by simple randomisation in a 1:1 ratio. Laboratory staff and researchers assessing outcomes were blinded to the treatment allocation.

All participants were sent 1 text message inviting them to get an STI test (see Box 1).

**Box 1. Wording of control and intervention text messages**

Control text message:

You have been invited to use a clinic-based sexual health service.

Please visit https://text4health.lshtm.ac.uk/trials/UI/public_htm/info/clinic.aspx to obtain your free STI test at a walk-in sexual health clinic.

If you have problems accessing this link, please text ‘HELP’

Intervention text message:

You have been invited to use an internet-based sexual health service.

Please visit https://sh24.org.uk/betatester to order your free STI test online.

Please do not share this link with anyone.

If you have problems accessing this link, please text ‘HELP’

If participants contacted the research team to say they had difficulty accessing the URL, they were resent the text message.

Participants in the intervention group were sent a text message with the URL of SH:24 (https://www.sh24.org.uk). SH:24 offers free postal self-sampling test kits for chlamydia, gonorrhoea, HIV, and syphilis. Participants who ordered a test kit from SH:24, were required to complete a short order form. Those reporting STI symptoms were advised via a pop-up message to visit their local clinic for immediate treatment. Those reporting complex needs such as depression, drug and alcohol dependency, or exploitative sexual partnerships were telephoned by a clinician and referred to relevant clinical services. All participants could continue to use the online service if they wished.

All test kits contained a lancet and collection tube to obtain a blood sample for serological testing for syphilis and HIV. For chlamydia and gonorrhoea, women were sent vaginal swabs and men were sent a container for first-catch urine samples. Test kits for MSM also contained swabs to take pharyngeal and rectal samples.

The tests kits included pictorial leaflets with guidance on how to collect the specimens. A video demonstrating blood sample collection was available on Youtube and could be accessed via the SH:24 website. Participants were kept informed of their order via text message. In the text messages, they were asked to contact the SH:24 team with any questions or concerns. After 2 weeks, non-returners were sent reminders via text and resent test kits if required, as per SH:24’s protocols.

Chlamydia, gonorrhoea, and syphilis test results were delivered by text message. Participants with reactive results for syphilis or positive results for chlamydia or gonorrhoea were signposted to local clinics for confirmatory testing and treatment as necessary. Reactive results for HIV were communicated by phone by a clinician.

Participants in the control group were sent the URL of a bespoke website with the contact details, websites, and locations (Google map images) of sexual health clinics in Lambeth and Southwark. These clinics provided usual care via walk-in services. Some clinics also offered an appointment service for those with symptoms or complex needs. Those diagnosed with an STI were asked to attend clinic for treatment. All participants were free to use any other sexual health services or interventions during the trial period. We used evidence-based methods to maximise response rates [29].

Our co-primary outcomes were self-reported diagnosis of an STI at 6 weeks, confirmed by patient health records, and self-reported completion of an STI test at 6 weeks, confirmed by patient health records. We defined completion of an STI test as samples processed by the laboratory and results delivered to SH:24 or to clinic.

Nucleic acid amplification tests (NAATs) were used to detect chlamydia and gonorrhoea in all services. In the online service, positive gonorrhoea results were confirmed using a second NAAT (Cepheid GeneXpert–Dual Target). Syphilis IgG/IgM was assessed (sensitivity > 99%; specificity > 99%), and HIV I and II/p24Ag were assessed (sensitivity 99.8–100%; specificity 99.9%). Reactive results for HIV or syphilis were counted as positive test results only when confirmed by assays in clinic. We defined STI diagnoses as those arising from laboratory testing.

Our secondary outcomes were the proportion of participants prescribed treatment for an STI, time from randomisation to completion of an STI test, and time from randomisation to treatment of an STI.

Our process outcomes were the proportion of STI tests that were positive in each group, median time from diagnosis to treatment in each group, the proportion of participants who completed an STI test in each group by service type, the proportion of participants diagnosed in each group by service type, and, in the intervention group only, the proportion who agreed that the intervention was acceptable and the proportion who adhered to an appropriate e-STI testing pathway. All pathways were considered appropriate unless participants completed a test via SH:24, received a negative result, and then retested for the same STI in a face-to-face setting within 6 weeks. In addition to our pre-specified process outcomes, we report the proportion of participants who tested positive for an STI among those who completed a test at 6 weeks, with 95% confidence intervals. All outcomes and their definitions are summarised in S1 Table.

Participants provided self-reported data by post or directly entered data on a website. Participants were sent £5 with a request to complete a follow-up questionnaire and an additional £5 on receipt of the completed questionnaire.

To obtain objective measures for our endpoints, we searched the SH:24 database, and data managers at the hospital trusts searched patient record databases, for all randomised participants using either (1) mobile phone and gender or (2) name and date of birth as identifiers. If participants reported using another service (general practitioner [GP] surgery or sexual health service outside of Lambeth and Southwark), we contacted the service to collect STI testing, diagnosis, and treatment data.

### Statistical analysis

The trial steering committee approved the pre-specified statistical analysis plan prior to unblinding. Our study was powered for our co-primary outcome of the proportion of participants diagnosed with an STI in each group [27,28]. Two factors determined the number of participants needed: the estimated proportion of participants with an STI and the size of the treatment effect.

We anticipated that not all of the intervention group would order a test kit. We estimated that 30% would not complete this first step. Among the 70% who ordered a kit, we assumed that 50% would return the kit for analysis (based on return rates of an e-STI testing service in the London borough of Greenwich).

There were no available data that would give us an estimate of the likely number of individuals who would complete an STI test in the control group. We assumed that fewer people (10%) would seek a test in clinic-based settings.

We based our STI prevalence estimates on the proportion of positive chlamydia tests among 15–24-year-olds in general practice settings in Lambeth and Southwark, which was 6% in 2012 [30]. We based our estimated loss to follow-up on previous e-health studies in the UK, which achieved 90% follow-up [31].

A sample size of 3,000 participants would lead to 90% power (2-sided alpha = 5%) to detect a relative risk (RR) of 3.5 (2.1% risk of diagnosis in the intervention group versus 0.6% risk of diagnosis in the control group), allowing for 10% loss to follow-up. This equates to 10% of the control group being tested, with a 6% probability of infection as in general practice settings, and 35% of the intervention group being tested, with a 6% probability of infection as in general practice settings.

With regard to our other co-primary outcome measure, 3,000 participants would lead to 99% power (2-sided alpha = 5%) to detect an absolute difference of 25% in the proportion of participants who completed a test in the intervention group versus the proportion who completed a test in the control group (35% versus 10%).

All analyses were undertaken on an intention to treat basis with Stata version 14.2. Effect measures were RRs with 95% confidence intervals and time to outcomes. We assessed overall heterogeneity for subgroups by summing the individual chi-squared statistics and their degrees of freedom to 1 overall chi-squared test on the sum of the degrees of freedom at a 5% level of significance.

For the primary analysis we used multivariate imputation by chained equations (MICE) to correct for any potential bias caused by missing data, assuming data are missing at random (MAR). Under this assumption, the distribution of the outcome for both missing and non-missing groups is the same for individuals with the same observed data. All baseline data were complete, except for sexual orientation, for which a missing category was used.

To obtain more precise estimates and confidence intervals with the correct coverage, we accounted for baseline factors by estimating the propensity score for randomised allocation for all participants [32]. We used a logistic regression model with randomised group as the response, and gender, age (years), number of sexual partners in the last 12 months, sexual orientation, and ethnicity as covariates.

We imputed our 2 co-primary outcomes (STI testing and STI diagnosis) and the secondary outcome proportion of participants prescribed treatment using 3 conditional models. Each imputation model included randomised group as a covariate and was weighted by the inverse of the estimated propensity score (for compatibility with the model for analysis). In addition, the 2 models to impute STI testing and STI diagnosis conditioned on self-reported testing, self-reported diagnosis, and self-reported treatment. The model to impute treatment conditioned on self-reported testing and self-reported treatment only, due to collinearity with other variables, which led to non-convergence. Each imputed data set was produced with 10 cycles. We generated 100 imputed data sets for each missing outcome. Multiple imputation inference proceeded via Rubin’s rules [33].

To explore departures from MAR assumptions for our co-primary outcomes, we performed a sensitivity analysis to explore the impact of possible differences between participants with complete outcome data and participants with missing outcome data. We multiply imputed missing outcome data, using inverse probability weighting on the estimated propensity score and with allocated group and self-reported testing, diagnosis, and treatment as covariates. The odds of STI diagnosis and the odds of a completed STI test for missing participants were varied to be 1/4, 1/2, 1, 2, and then 4 times as large as the MAR analyses; this was done factorially for the 2 randomised groups, giving a total of 25 analyses (including the principal analysis assuming MAR).

We explored heterogeneity of the intervention effect on our primary outcomes. We tested for interaction at a 5% level of significance to assess whether effectiveness varied by gender (male, female), ethnicity (white, black/African/Caribbean/black British, Asian/Asian British/all other groups), sexual orientation (MSM, all other groups), age group (16–19 years, 20–24 years, 25–30 years), number of sexual partners (1, 2+), SH:24 availability (period when available to study participants only, period when available to all residents in Lambeth and Southwark), and Index of Multiple Deprivation rank (linear). The Index of Multiple Deprivation is a relative measure of deprivation that ranks every small area in England from 1 (most deprived area) to 32,844 (least deprived area) [34].

These analyses were conducted in the complete cases under a MAR assumption. They were not weighted by the inverse of the estimated propensity score, as specified in the analysis plan, due to non-convergence of the models.

We used survival analysis to estimate time from randomisation to test completion and time from randomisation to treatment. We estimated the restricted mean survival time (RMST), which is a meaningful measure even when the proportional hazards assumption is in doubt. As with other analyses, the RMST accounted for covariates by weighting on the inverse of the estimated propensity score. We set the restricted mean time t* = 6 weeks (42 days) for time to test and t* = 3 months (84 days) for time to treatment using a ‘3df/1df’ Royston–Parmar model [35].

## Results

**Fig 1:**
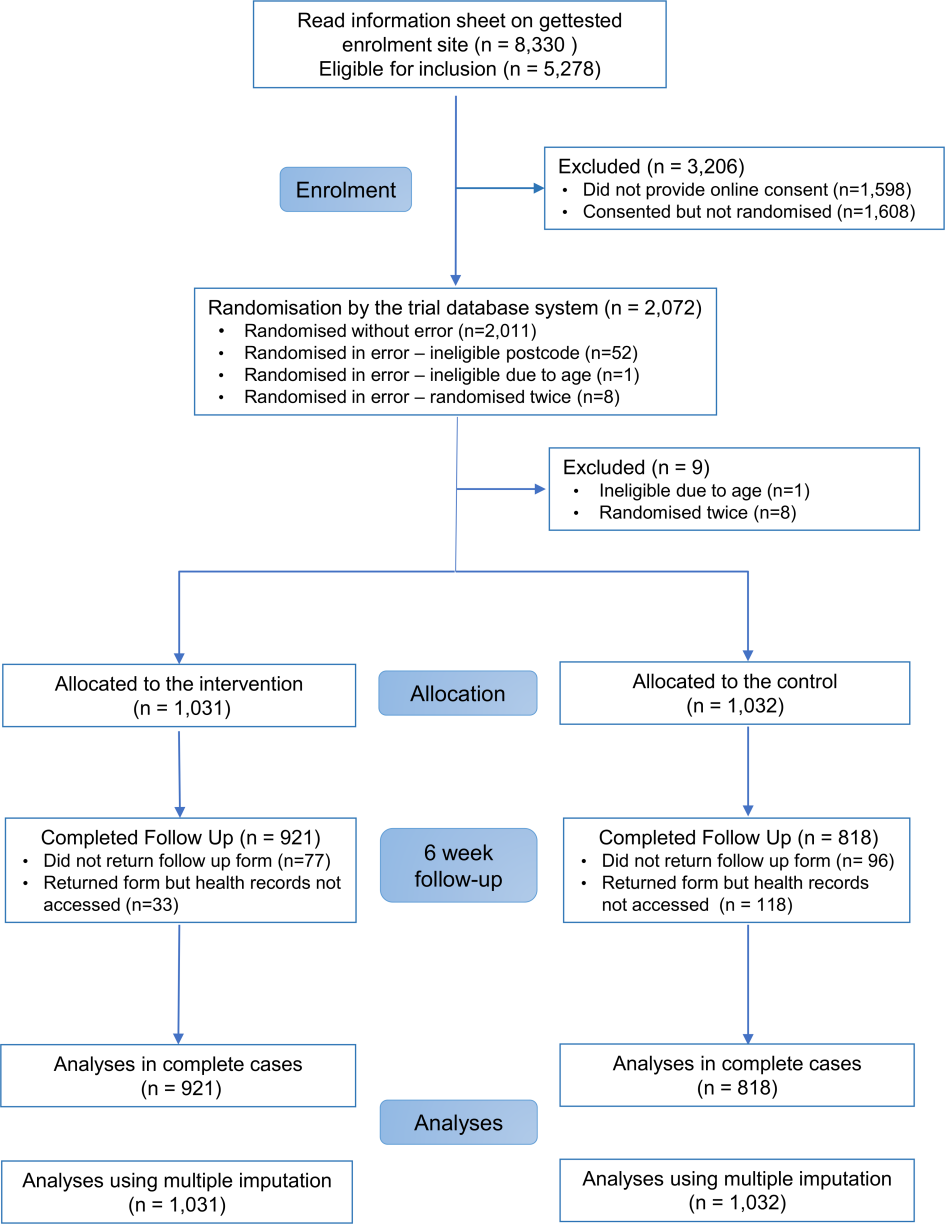
CONSORT flow diagram.

In all, 2,072 participants were randomly assigned to the SH:24 online testing and results service or to the control group (Fig 1). We excluded 8 participants who were randomised twice and 1 participant who was randomised and did not meet the age criterion (Fig 1). We were unable recruit to target, and therefore we lacked power for the co-primary outcome of STI diagnoses. Baseline characteristics are presented in Table 1.

**Table 1:** Baseline characteristics of participants.

Characteristic	Intervention group (*n* = 1,031)	Control group (*n* = 1,032)
**Gender**		
Female	604 (58.6%)	609 (59.0%)
Male	424 (41.1%)	422 (40.9%)
Transgender	3 (<1.0%)	1 (<1.0%)
**Mean age (years)**	23 (3.5)	23 (3.6)
**Age group (years)**		
16–19	206 (20.0%)	220 (21.3%)
20–24	440 (42.7%)	432 (41.9%)
25–30	385 (37.3%)	380 (36.8%)
**Sexual orientation**		
Men who have sex with men	129 (12.5%)	133 (12.9%)
Other	890 (86.3%)	888 (86.0%)
Refused	12 (1.2%)	11 (1.1%)
**Number of partners**		
1	302 (29.3%)	304 (29.5%)
2+	729 (70.7%)	728 (70.5%)
**Ethnic group**		
White	779 (75.6%)	749 (72.6%)
Black/African/Caribbean/black British	81 (7.9%)	110 (10.7%)
Asian/Asian British	70 (6.8%)	57 (5.5%)
Mixed/multiple ethnicity	89 (8.6%)	99 (9.6%)
Other	12 (1.2%)	17 (1.6%)
**Last STI test (months)**		
0–3	144 (14.0%)	155 (15.0%)
3–6	161 (15.6%)	140 (13.6%)
6–12	181 (17.6%)	165 (16.0%)
12+	301 (29.2%)	288 (27.9%)
Never	244 (23.7%)	284 (27.5%)
**Place of last STI test**		
Sexual health clinic	521 (50.5%)	494 (47.9%)
General practice	121 (11.7%)	115 (11.1%)
Hospital	51 (4.9%)	43 (4.2%)
Pharmacy	7 (0.7%)	11 (1.1%)
Internet STI test	32 (3.1%)	28 (2.7%)
Other	55 (5.3%)	55 (5.3%)
Not applicable/not available	244 (23.7%)	286 (27.7%)

Data are *n* (%) or mean (SD).

STI, sexually transmitted infection.

Primary outcome data, prior to multiple imputation, were available for 921 (89%) participants in the intervention group and 818 (79%) in the control group (Fig 1).

The proportions of participants in each group who reported completing a test at 6 weeks, and who were confirmed to have tested via patient record checks, are provided in S1 Fig. Record checks in clinics, SH:24, and GP surgeries were completed by 17 June 2016.

Our primary analyses were based on multiply imputed data sets. In all, 1,031 in the intervention group and 1,032 in the control group were included in the analyses. At 6 weeks, 50.0% of the intervention group had completed an STI test compared to 26.6% in the control group (RR 1.87, 95% CI 1.63 to 2.15, *P <* 0.001; Table 2); 2.8% of the intervention group versus 1.4% in the control group had been diagnosed with an STI (RR 2.10, 95% CI 0.94 to 4.70, *P =* 0.079; Table 2).

**Table 2:** Primary and secondary outcomes.

Outcome	Intervention (*n* = 1,031)	Control (*n* = 1,032)	Risk difference (95% CI)	Relative risk (95% CI)	*P* value
**Primary outcomes (MICE)**					
Diagnosis of STI at 6 weeks	2.8%	1.4%	1.4% (−0.1, 3.1)	2.10 (0.94, 4.70)	0.079
Completion of STI test at 6 weeks	50.0%	26.6%	23.2% (18.7, 27.8)	1.87 (1.63, 2.15)	<0.001
**Secondary outcome (MICE)**					
STI cases treated	1.1%	0.7%	0.8% (−0.5, 2.1)	1.72 (0.71, 4.16)	0.231

All estimates (including proportions) are derived from multiply imputed data sets.

MICE, multivariate imputation by chained equations (number of imputations = 100); STI, sexually transmitted infection.

We obtained similar results for the complete case analysis (S2 Table) and for all the scenarios that we investigated under the assumption that our missing outcome data were missing not at random (S2 and S3 Figs).

The proportion of participants treated was 1.1% in the intervention group versus 0.7% in the control group (RR 1.72, 95% CI 0.71 to 4.16, *P =* 0.231; Table 2). In the complete cases, time to test, estimated by the RMST, was shorter in the intervention group compared to the control group (28.8 days versus 36.5 days, *P <* 0.001; Table 3); no differences were observed for time to treatment (83.2 days versus 83.5 days, *P =* 0.51; Table 3).

**Table 3:** Secondary outcomes (time to event).

Secondary outcome	RMST (SE)		RMST difference (95% CI)	*P* value
	Intervention	Control		
Time to test (t* = 42 days)	28.8 (0.5)	36.5 (0.4)	7.7 days (6.4, 8.9)	<0.001
Time to treatment (t* = 84 days)	83.2 (0.3)	83.5 (0.2)	0.3 days (−0.6, 1.2)	0.51

Estimates derived from complete cases.

RMST, restricted mean survival time.

**Fig 2:**
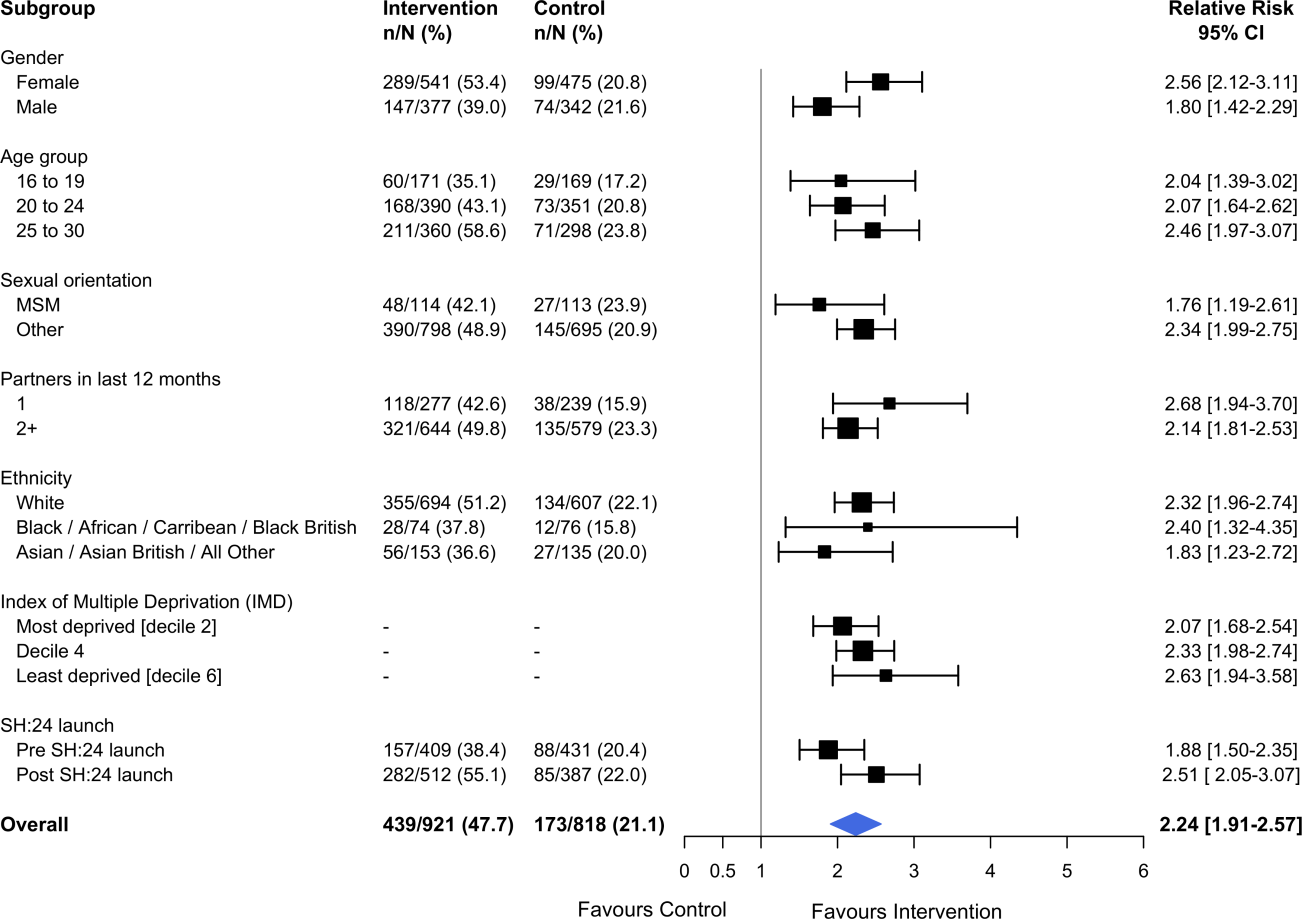
Effect of the SH:24 intervention on STI testing by subgroup. Interaction test: chi-squared = 12.36 on 9 degrees of freedom, *P =* 0.19. Estimates derived from the complete cases. MSM, men who have sex with men; STI, sexually transmitted infection.

**Fig 3:**
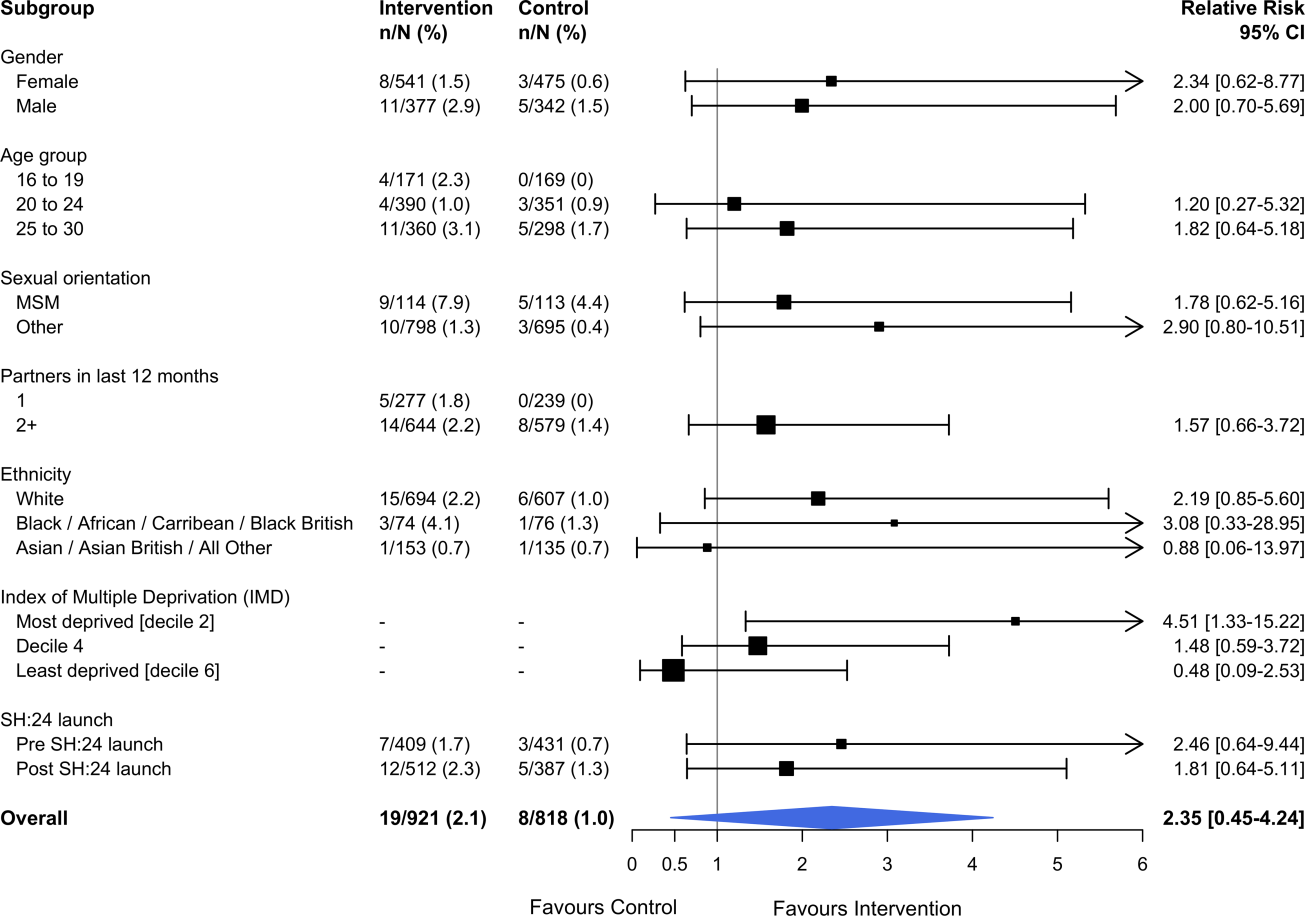
Effect of the SH:24 intervention on STI diagnoses by subgroup. Interaction test: chi-squared = 4.57 on 5 degrees of freedom, *P =* 0.46. Estimates derived from the complete cases. MSM, men who have sex with men; STI, sexually transmitted infection.

Kaplan–Meier plots for time to test and time to treatment are presented in S4 Fig and S5 Fig. We identified no evidence of heterogeneity for any of the pre-specified subgroup analyses, which were conducted in the complete cases (Figs 2 and 3). Given that we lacked power for the analyses of STI diagnoses, the subgroup analyses for this outcome are even more underpowered (Fig 3).

### Process outcomes

Among participants who completed an STI test at 6 weeks, 4.3% (19/439; 95% CI 2.8 to 6.7) tested positive for an STI in the intervention group and 4.6% (8/173; 95% CI 2.3 to 9.0) tested positive for an STI in the control group. The median time from diagnosis to treatment among those with complete treatment data was 2 days in the intervention group and 4 days in the control group (S3 Table). Four of the 18 cases treated received treatment prior to a laboratory-confirmed diagnosis and were not included in these summary statistics. The excluded cases were evenly distributed between groups.

Three participants in the intervention group tested via SH:24, received a negative result, and retested in clinic for the same STI within 6 weeks (S3 Table).

Regarding intervention acceptability, 76% (294/388) of participants in the intervention group who tested via SH:24 provided acceptability data. Of these, 71% (209/294) found the intervention to be acceptable (S3 Table).

In all, 88% (388/439) of participants in the intervention group who completed an STI test at 6 weeks tested via SH:24 (Table 4). Nineteen participants in the intervention group were diagnosed with an STI. Of these, 12 were diagnosed via SH:24 and 7 were diagnosed in a sexual health clinic (Table 4).

**Table 4:** STI test completion and STI diagnoses by service type.

Service type	STI test completion		STI diagnosis	
	Intervention	Control	Intervention	Control
Sexual health clinic in Lambeth or Southwark	41 (9%)	145 (84%)	3 (16%)	4 (50%)
Other sexual health clinic	9 (2%)	15 (9%)	4 (21%)	3 (38%)
General practice	1 (<1%)	2 (1%)	0 (0%)	0 (0%)
SH:24	388 (88%)	11 (6%)	12 (63%)	1 (13%)
Total	439 (100%)	173 (100%)	19 (100%)	8 (100%)

Data are *n* (%).

STI, sexually transmitted infection.

In the intervention group, 2.8% (12/432) of chlamydia tests were positive, 1.4% (6/433) of gonorrhoea tests were positive, and 0.8% (3/363) of syphilis tests were positive. In the control group, 2.4% (4/169) of chlamydia tests were positive, 3% (5/169) of gonorrhoea tests were positive, and none of the 137 (0/137) syphilis tests were positive.

In all, 365 participants tested for HIV in the intervention group, and 140 in the control group. There were no confirmed HIV diagnoses. One participant tested for hepatitis B in the intervention group, and 11 in the control group. There were no hepatitis B diagnoses in either group.

Among participants who were diagnosed with an STI, most (24/27) were diagnosed with a single STI, and 3 with more than 1 STI.

## Discussion

### Statement of principal findings

When STI testing is promoted, offering e-STI testing alongside usual care significantly increases uptake of STI testing. The intervention increased STI testing in all groups including those at high risk for STIs. We lacked power for the analyses of STI diagnoses and STI cases treated, but our estimates are in the expected direction. The intervention reduced time to test but not time to treatment.

### Strengths and weaknesses

This study has several strengths. We used an independent, remote computer-based randomisation system to ensure study staff had no prior knowledge of the treatment allocation. We collected objective outcomes even for those participants who tested outside of Lambeth and Southwark or via a different pathway (e.g., at their GP). Laboratory staff and researchers carrying out the analyses were blinded to the allocation. Baseline prognostic factors were well balanced between groups, and our co-primary outcomes were known for 84% of participants. In trials where it is not possible to blind participants, allocation to the control group could reduce motivation to carry out the desired behaviour. To mitigate potential performance bias, participants were informed at the time of recruitment that they would be invited via text message to use one type of sexual health service without stating the options. All analyses were intention to treat.

We successfully recruited high-risk groups including MSM (262/2,063; 13%), 16–24-year-olds (1,298/2,063; 63%), and individuals reporting 2 or more sexual partners in the last year (1,457/2,063; 71%). We enrolled individuals who reported limited prior contact with conventional STI testing services. A quarter of our study population had never tested for STIs prior to the trial (528/2,063) (Table 1). In all, 17% of MSM (45/262) and 21% of 16–24-year-olds (277/1,298) had not tested within the last 12 months, despite national guidelines that recommend annual STI testing among these groups [36,37].

Our trial has a number of limitations. We fell short of our recruitment target of 3,000 participants, and we were unable to extend the recruitment period due to a pre-existing plan to promote SH:24 widely across the study area. As a result, the study lacked power to detect differences in STI diagnoses and STI cases treated. As with other online enrolment systems [38], a high number of potential participants started, but did not complete, the enrolment process (Fig 1).

It is likely that those who enrolled in the study had a greater interest in STI testing than those who declined. Testing uptake in the control and intervention groups in all trials may be higher than in the general population. This could result in a smaller risk difference if the intervention were to be implemented in the general population. In all, 26% of our study population identified as BME at baseline. This is lower than the proportion of individuals in Lambeth and Southwark identifying as BME (44% and 48%, respectively [39,40]), which may limit the generalisability of our results.

While there is some reporting bias in the self-reported data (S1 Fig), the potential to bias our co-primary endpoints is limited, as these were objectively verified via participants’ health records. There was potential for contamination as the URL for SH:24 was promoted in Lambeth and Southwark when the service was launched in March 2015. However, only 11 control group participants used SH:24 in the trial, and any contamination would have biased our results towards the null. In our subgroup analyses, there was no evidence of heterogeneity as a result of SH:24’s change in availability.

At enrolment, participants were informed that the £10 incentive was for completing follow-up, but some participants later reported that they thought it was for completing an STI test. Given that all participants were told about the £10 incentive, and sent money at follow-up, the impact of this incentivisation should be non-differential and would not explain our statistically significant results.

Although the laboratory tests used by services are highly sensitive and specific, some misclassification is possible. This misclassification could have biased our STI diagnosis results towards the null. We randomised 8 people twice and excluded them from the analyses. It is possible that we randomised other people twice but only if they provided an incorrect name and date of birth. All service providers were motivated to record STI testing data in line with national surveillance requirements. If some providers were more accurate than others, this might result in differential misclassification and bias.

Although we achieved high response rates for our co-primary outcomes, these rates were differential as we achieved higher follow-up in the intervention group than in the control group. This can result in biased estimates under a complete case approach [41]. To deal with missing outcome data, our primary analyses used multivariate imputation techniques under the assumption that data were MAR. This approach is well established, and it is more valid and efficient than other approaches to deal with missing data [42]. It is reassuring that the results of all sensitivity analyses were similar to the results for the primary analyses.

### Strengths and weaknesses in relation to other studies

To our knowledge, this is the first trial of an e-STI testing service that offers testing for 4 STIs (chlamydia, gonorrhoea, HIV, and syphilis). Descriptive studies from the US suggest that services that offer internet-based testing for chlamydia, gonorrhoea, and trichomoniasis can attract at-risk populations (young and BME groups) and yield high STI positivity [43,44]. One randomised controlled trial in France has evaluated self-sampling for chlamydia accessed via the internet compared to chlamydia testing in face-to-face settings. It reports an increase in testing uptake (29.2% in the intervention group versus 8.7% in the control group, RR 3.37, 95% CI 3.05 to 3.74). However, outcomes were assessed using different measures in the intervention and control groups, and there was low and differential follow-up (47% follow-up in the intervention group versus 30% follow-up in the control group) [45].

Our finding of increased STI testing uptake with e-STI testing is similar to increases in testing reported in trials of self-sampling and self-testing interventions that are not accessed via the internet [46,47].

### Meaning and mechanisms

The theory of change underpinning SH:24 proposes that online diagnostic pathways will increase testing uptake as they are convenient, private, and non-judgemental and offer more choice than traditional clinic pathways [17].

The similar effect of e-STI testing on STI testing uptake across different population groups is of public health importance as it suggests a potential to increase testing among those most in need. Moreover, higher proportions of participants in the intervention group tested for all infections, including HIV and syphilis, compared to the control group.

Seven of the 19 participants diagnosed in the intervention group were diagnosed in a sexual health clinic. This highlights the continued importance of face-to-face services and is consistent with the theory of change, which proposes that e-STI testing offers patients more choice.

While our findings are in line with the proposed theory of change, they provide little evidence regarding the mechanism of action. Qualitative research is underway to explore participants’ experiences of using the intervention and their views on how it may have worked. These findings will be reported separately.

National guidelines in the UK recommend increasing testing among key population groups, and in areas of high HIV prevalence, in order to detect asymptomatic infection and normalise testing practices [18,19]. Our results for STI testing uptake suggest that e-STI testing could play a role in achieving these public health objectives. STI testing uptake and STI diagnosis are important intermediary outcomes on the pathway to increasing cases treated and cured or managed in community settings. However, our study provides limited evidence on these latter outcomes.

e-STI testing is currently being implemented in the UK as one measure to meet increasing demand for STI testing against a backdrop of severe budget cuts [48,49]. Publication of the cost-effectiveness evaluation of the intervention is pending and may provide additional insights on the contribution of e-STI testing. A larger trial is required to assess outcomes later in the cascade of STI care including STI diagnoses and cases treated and cured.

The effect size for STI cases treated was lower than for STI diagnoses, as we were unable to confirm if all those diagnosed were treated. At the time of the trial, SH:24 required those diagnosed with an infection to attend clinics in person for treatment. Attendance at clinic for treatment was confirmed for 11 of 19 participants diagnosed with an STI in the intervention group and 7 of 8 participants diagnosed in the control group. Some participants may have obtained treatment outside the study area, but as they did not state where they were treated, we were unable to verify this. The intervention did not reduce time to treatment.

It is plausible that whilst the intervention removed the barrier of having to attend a clinical service for testing, the subsequent requirement to attend clinic for treatment may have deterred some participants. Additional inputs are required so that increases in STI testing and STI diagnoses translate into similar increases in cases treated. This is likely to ensure that the potential public health benefits of e-STI testing can be fully realised.

## Conclusions

We trialled SH:24 in a community setting, in 2 boroughs well served by face-to-face clinical services. Providing e-STI testing in contexts where supply is more limited, or targeting particular high-risk groups, might strengthen the contribution of e-STI testing to the control and management of STIs.

e-STI testing models could be adapted for countries with sufficient laboratory facilities. Established distribution channels for health products may be suitable for sending and receiving test kits, where postal services are limited. Future iterations of e-STI testing could include a wider range of services such as self-testing for HIV. Self-testing differs from the self-sampling modality evaluated by this study. Self-testing enables individuals to take a sample, perform a test, and interpret the results by themselves, without the need of a laboratory [14].

Delivering e-STI testing and results services to scale is technically feasible as demonstrated by SH:24, which currently delivers 42,000 tests per year in 6 regions in the UK. The long-term public health benefits of e-STI services will depend on testing, diagnosis, and treatment rates when implemented. These outcomes should be subject to ongoing monitoring and evaluation.

## Supporting information

S1 Data (XLS)

S1 Fig Confirmation of self-reported responses. (PPTX)

S2 Fig Sensitivity analysis: Completion of STI test at 6 weeks. (PDF)

S3 Fig Sensitivity analysis: STI diagnosis at 6 weeks. (PDF)

S4 Fig Kaplan–Meier plot: Time to STI test. (PDF)

S5 Fig Kaplan–Meier plot: Time to STI treatment. (PDF)

S1 Table Summary of outcomes. (DOCX)

S2 Table Primary and secondary outcomes. Comparison of analyses based on multiple imputation (MI) and analyses in the complete cases.
(PPTX)

S3 Table Process outcomes. (PPTX)

S1 Text Trial protocol. (PDF)

S2 Text Trial protocol addendum. (PDF)

S3 Text CONSORT checklist. (DOC)

## Abbreviations

BME: black and minority ethnic
e-STI testing: internet-accessed sexually transmitted infection testing
GP: general practitioner
MAR: missing at random
MSM: men who have sex with men
RMST: restricted mean survival time
RR: relative risk
SMS: short message service
STI: sexually transmitted infection
URL: uniform resource locator
