# Supplementary material for: Internet-accessed sexually transmitted infection (e-STI) testing and results service: A randomised, single-blind, controlled trial
Source: PLoS Med. 2017 Dec 27;14(12):e1002479. doi: 10.1371/journal.pmed.1002479 (PMC5744909; doi:10.1371/journal.pmed.1002479)
Supplement: S1 Fig — (PPTX) [file pmed.1002479.s002.pptx]

## Slide 1
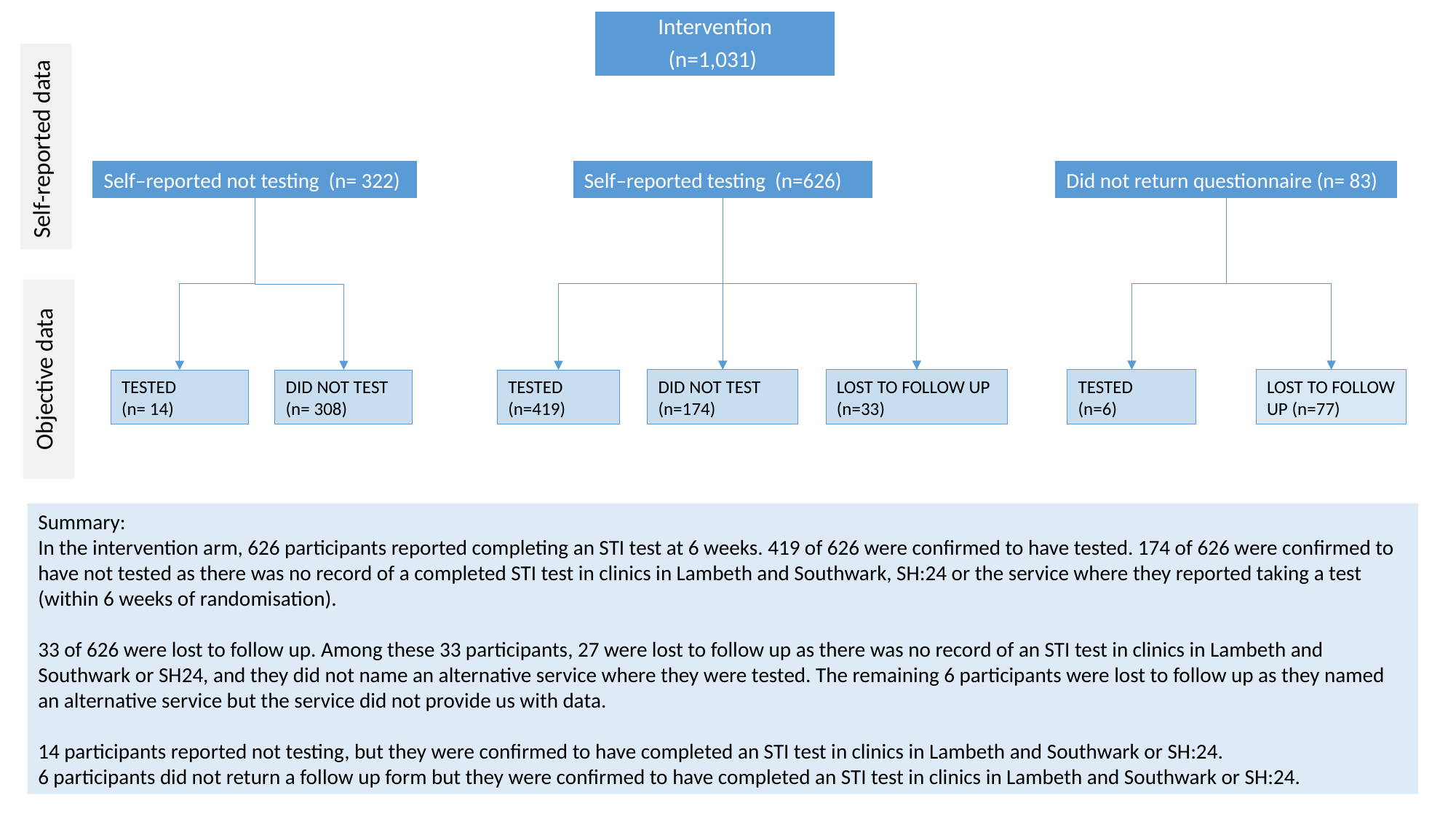

Self-reported data
Self–reported not testing (n= 322)
Self–reported testing (n=626)
Did not return questionnaire (n= 83)
Objective data
LOST TO FOLLOW UP (n=77)
TESTED
(n=6)
DID NOT TEST (n=174)
LOST TO FOLLOW UP (n=33)
TESTED (n=419)
DID NOT TEST
(n= 308)
TESTED
(n= 14)
Summary:
In the intervention arm, 626 participants reported completing an STI test at 6 weeks. 419 of 626 were confirmed to have tested. 174 of 626 were confirmed to have not tested as there was no record of a completed STI test in clinics in Lambeth and Southwark, SH:24 or the service where they reported taking a test (within 6 weeks of randomisation).
33 of 626 were lost to follow up. Among these 33 participants, 27 were lost to follow up as there was no record of an STI test in clinics in Lambeth and Southwark or SH24, and they did not name an alternative service where they were tested. The remaining 6 participants were lost to follow up as they named an alternative service but the service did not provide us with data.
14 participants reported not testing, but they were confirmed to have completed an STI test in clinics in Lambeth and Southwark or SH:24.
6 participants did not return a follow up form but they were confirmed to have completed an STI test in clinics in Lambeth and Southwark or SH:24.

## Slide 2
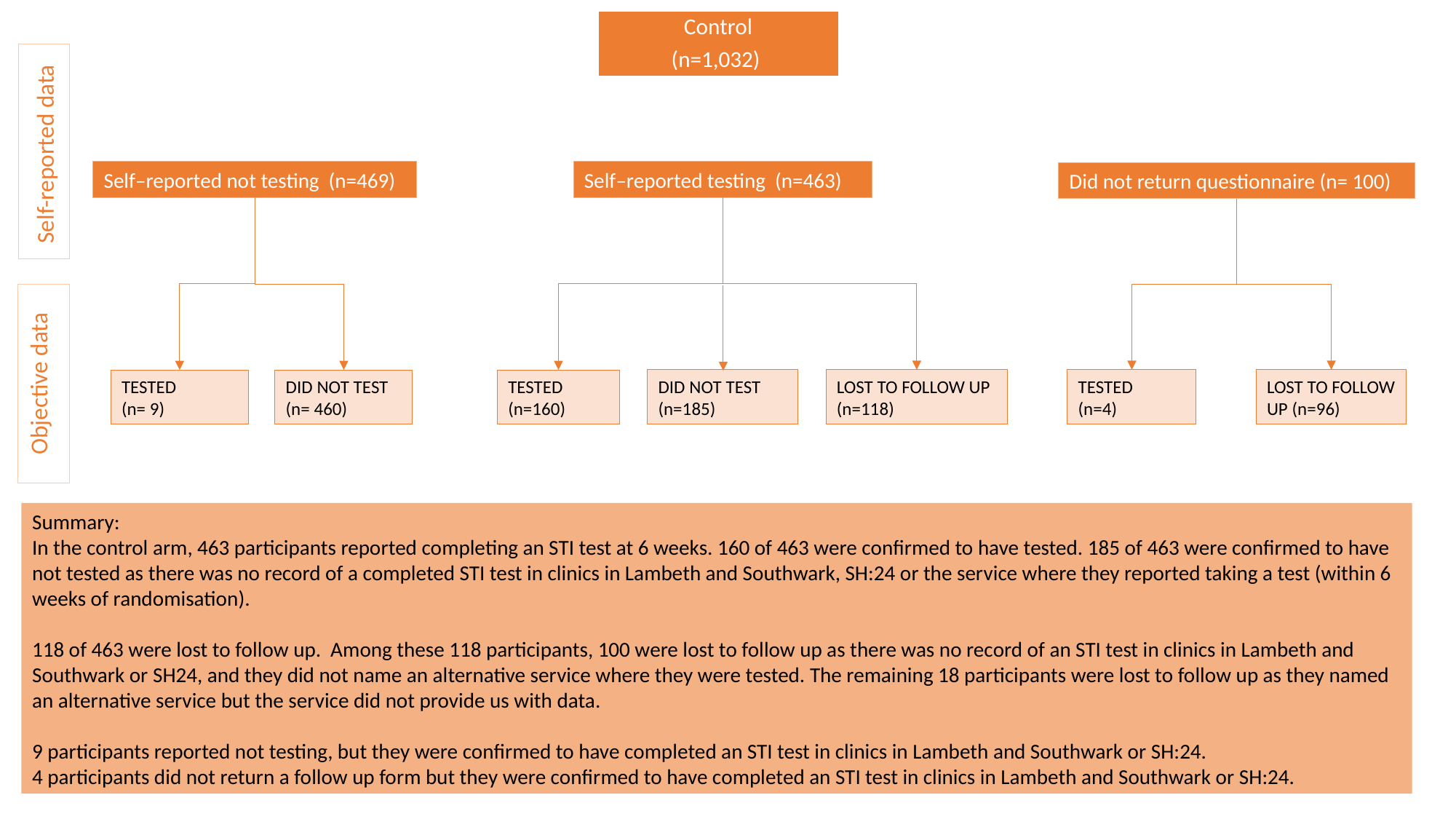

Self-reported data
Self–reported not testing (n=469)
Self–reported testing (n=463)
Did not return questionnaire (n= 100)
Objective data
LOST TO FOLLOW UP (n=96)
TESTED
(n=4)
DID NOT TEST (n=185)
LOST TO FOLLOW UP (n=118)
TESTED (n=160)
DID NOT TEST
(n= 460)
TESTED
(n= 9)
Summary:
In the control arm, 463 participants reported completing an STI test at 6 weeks. 160 of 463 were confirmed to have tested. 185 of 463 were confirmed to have not tested as there was no record of a completed STI test in clinics in Lambeth and Southwark, SH:24 or the service where they reported taking a test (within 6 weeks of randomisation).
118 of 463 were lost to follow up. Among these 118 participants, 100 were lost to follow up as there was no record of an STI test in clinics in Lambeth and Southwark or SH24, and they did not name an alternative service where they were tested. The remaining 18 participants were lost to follow up as they named an alternative service but the service did not provide us with data.
9 participants reported not testing, but they were confirmed to have completed an STI test in clinics in Lambeth and Southwark or SH:24.
4 participants did not return a follow up form but they were confirmed to have completed an STI test in clinics in Lambeth and Southwark or SH:24.
