# Supplementary figures and images for: Internet-accessed sexually transmitted infection (e-STI) testing and results service: A randomised, single-blind, controlled trial
Source: PLoS Med. 2017 Dec 27;14(12):e1002479. doi: 10.1371/journal.pmed.1002479 (PMC5744909; doi:10.1371/journal.pmed.1002479)

MNAR imputation odds ratio  
(Control, Intervention)

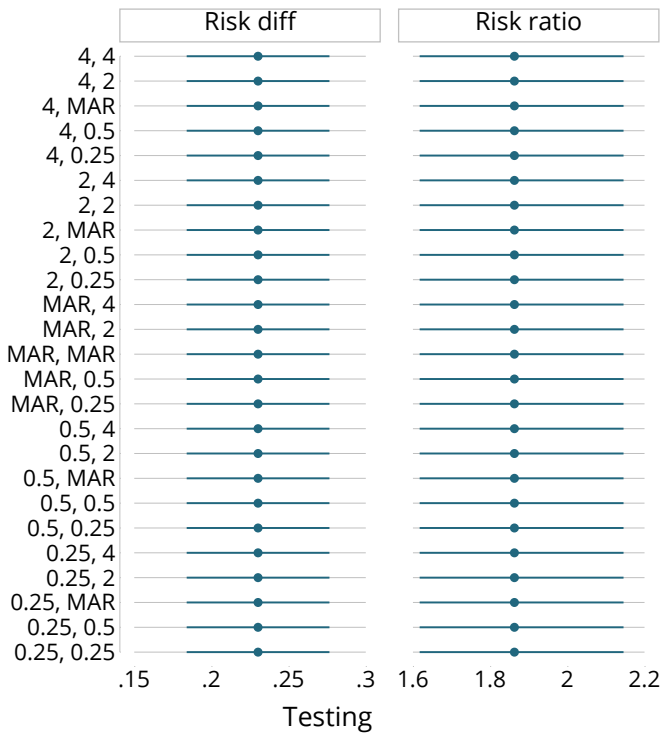

Supplement: S2 Fig — (PDF) [file pmed.1002479.s003.pdf]

MNAR imputation odds ratio  
(Control, Intervention)

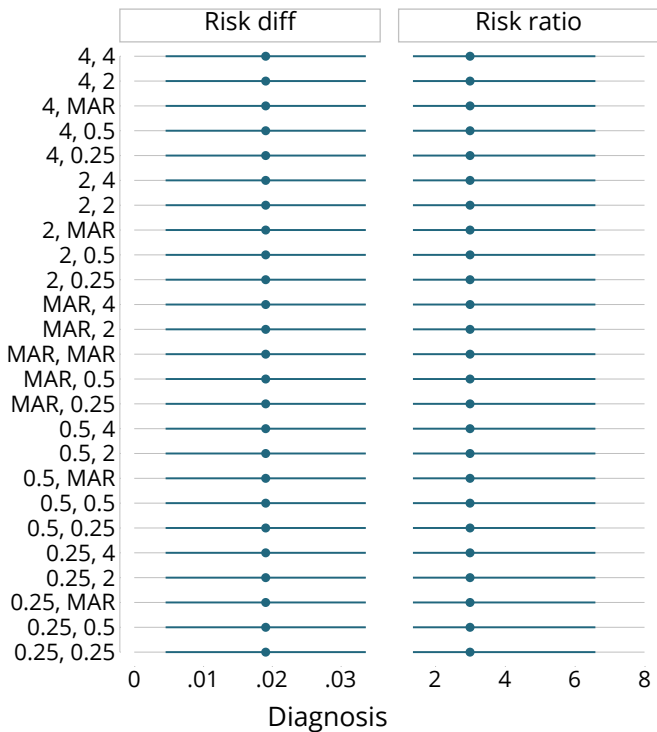

Supplement: S3 Fig — (PDF) [file pmed.1002479.s004.pdf]

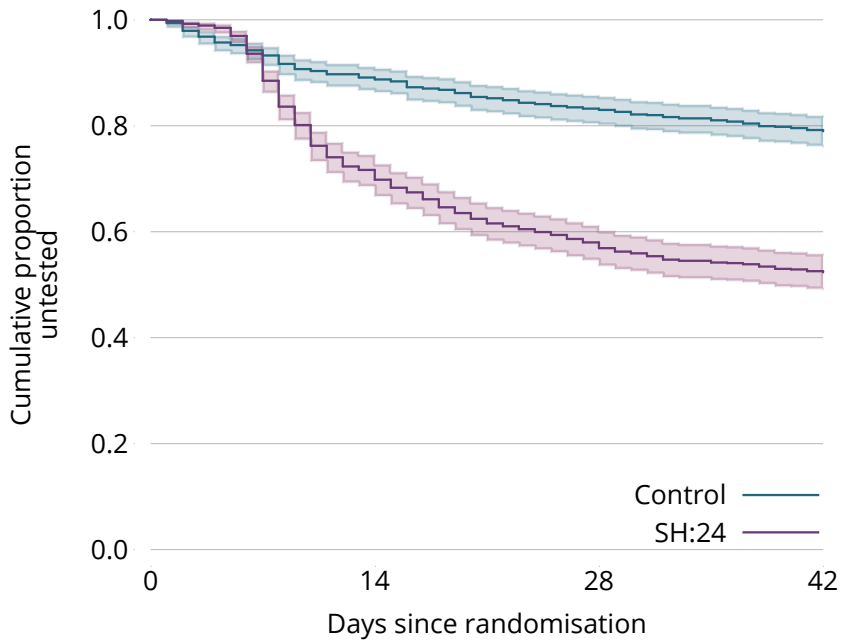

No. remaining

Control 817

SH:24 921

770

862

728

660

698

575

680

534

665

502

647

484

Supplement: S4 Fig — (PDF) [file pmed.1002479.s005.pdf]

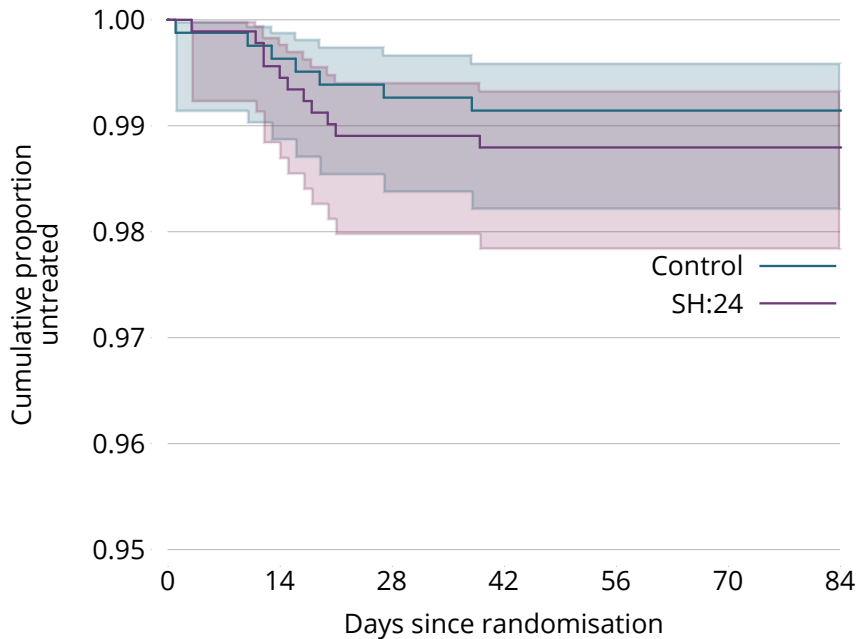

No. remaining

Control 817

SH:24 913

814

909

811

903

810

902

810

902

810

902

810

902

Supplement: S5 Fig — (PDF) [file pmed.1002479.s006.pdf]
