# Supplementary material for: Internet-accessed sexually transmitted infection (e-STI) testing and results service: A randomised, single-blind, controlled trial
Source: PLoS Med. 2017 Dec 27;14(12):e1002479. doi: 10.1371/journal.pmed.1002479 (PMC5744909; doi:10.1371/journal.pmed.1002479)
Supplement: S1 Table — (DOCX) [file pmed.1002479.s007.docx]

| Outcome | Definition | Measure of Effect |
| --- | --- | --- |
| Primary Outcomes |  |  |
| Uptake of STI testing at 6 weeks | Proportion of participants in each arm who completed an STI test at 6 weeks | Relative Risk, 95% CI |
| STI cases diagnosed | Proportion of participants in each arm who were diagnosed with an STI | Relative Risk, 95% CI |
| Secondary Outcomes |  |  |
| STI cases treated | Proportion of participants in each arm prescribed treatment for an STI | Relative Risk, 95% CI |
| Time to test | Restricted mean survival time (days) from randomisation to completion of an STI test, in each arm | Restricted Mean Survival Time Difference, 95% CI |
| Time to treatment | Restricted mean survival time (days) from randomisation to treatment, in each arm | Restricted Mean Survival Time Difference, 95% CI |
| Process Outcomes (summarised in each arm) | | |
| STI positivity | Among those who completed an STI test at 6 weeks, the proportion who tested positive for an STI | N/A |
| STI test positivity | The proportion of STI tests that were positive (for each infection) | N/A |
| Time from diagnosis to treatment | Among those with complete treatment data, the median time (days) from diagnosis to treatment | N/A |
| Uptake of testing by service type | Service where completed an STI test (among participants who tested) | N/A |
| STI diagnoses by service type | Service where diagnosed (among participants who were diagnosed) | N/A |
| Process Outcomes (summarised in the intervention arm only) | |  |
| Acceptability of the intervention | Among those who provided acceptability data in the intervention arm, the proportion who found the intervention to be acceptable  ** Acceptability was constructed as a binary variable (see S3 Table for details)* | N/A |
| Adherence to SH:24 testing pathway | Among those who tested via SH:24 in the intervention arm, the proportion who adhered to the SH:24 testing pathway * *All pathways deemed acceptable unless a participant tested via SH:24, received a negative result and tested for the same STI in clinic settings, within 6 weeks* | N/A |

**S1 Table**.
