# Supplementary material for: Internet-accessed sexually transmitted infection (e-STI) testing and results service: A randomised, single-blind, controlled trial
Source: PLoS Med. 2017 Dec 27;14(12):e1002479. doi: 10.1371/journal.pmed.1002479 (PMC5744909; doi:10.1371/journal.pmed.1002479)
Supplement: S2 Table — Comparison of analyses based on multiple imputation (MI) and analyses in the complete cases. (PPTX) [file pmed.1002479.s008.pptx]

## Slide 1
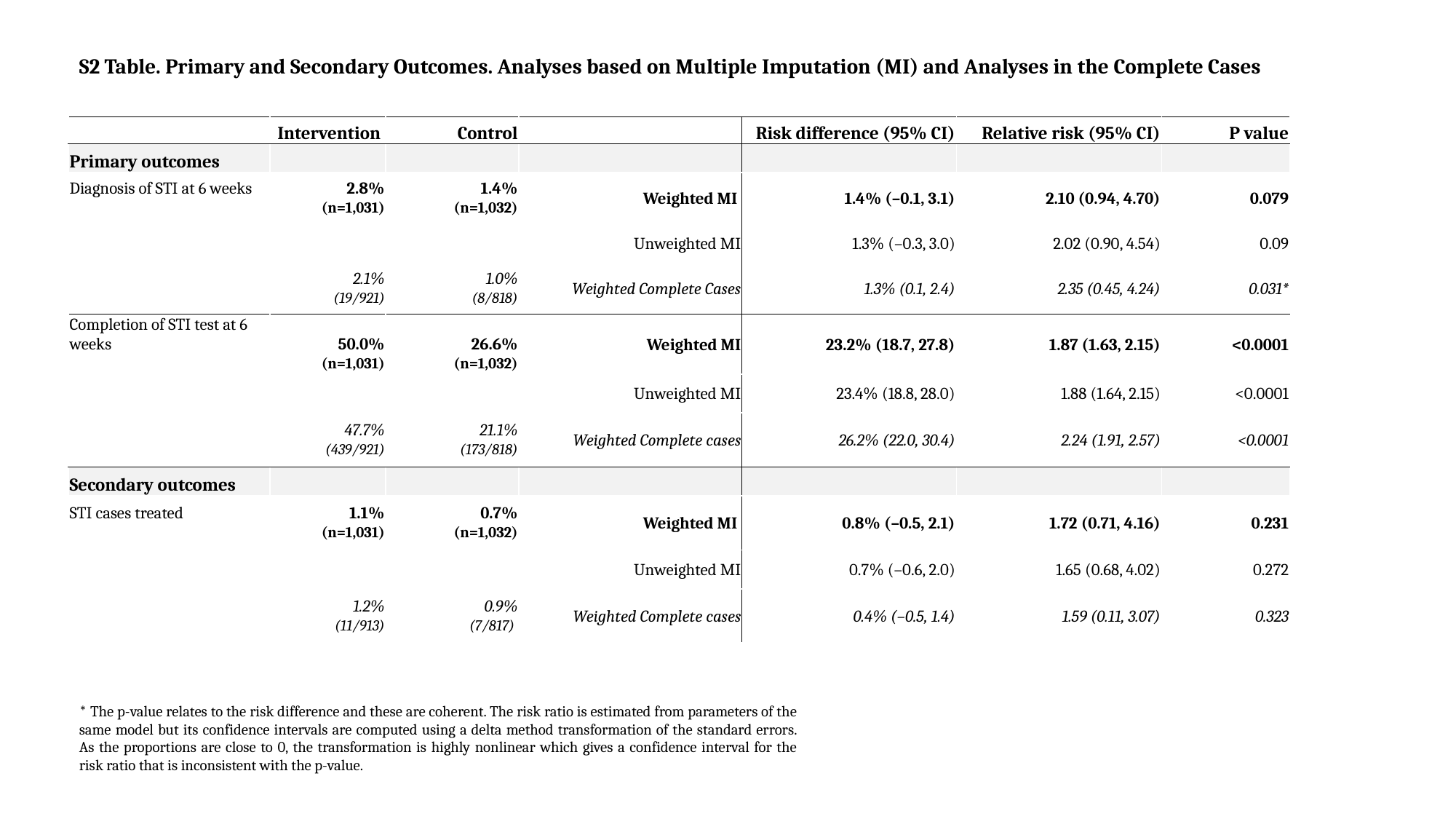

S2 Table. Primary and Secondary Outcomes. Analyses based on Multiple Imputation (MI) and Analyses in the Complete Cases
| | Intervention | Control | | Risk difference (95% CI) | Relative risk (95% CI) | P value |
| --- | --- | --- | --- | --- | --- | --- |
| Primary outcomes | | | | | | |
| Diagnosis of STI at 6 weeks | 2.8% | 1.4% | Weighted MI | 1.4% (–0.1, 3.1) | 2.10 (0.94, 4.70) | 0.079 |
| | (n=1,031) | (n=1,032) | | | | |
| | | | Unweighted MI | 1.3% (–0.3, 3.0) | 2.02 (0.90, 4.54) | 0.09 |
| | 2.1% | 1.0% | Weighted Complete Cases | 1.3% (0.1, 2.4) | 2.35 (0.45, 4.24) | 0.031\* |
| | (19/921) | (8/818) | | | | |
| Completion of STI test at 6 weeks | 50.0% | 26.6% | Weighted MI | 23.2% (18.7, 27.8) | 1.87 (1.63, 2.15) | <0.0001 |
| | (n=1,031) | (n=1,032) | | | | |
| | | | Unweighted MI | 23.4% (18.8, 28.0) | 1.88 (1.64, 2.15) | <0.0001 |
| | 47.7% | 21.1% | Weighted Complete cases | 26.2% (22.0, 30.4) | 2.24 (1.91, 2.57) | <0.0001 |
| | (439/921) | (173/818) | | | | |
| Secondary outcomes | | | | | | |
| STI cases treated | 1.1% | 0.7% | Weighted MI | 0.8% (–0.5, 2.1) | 1.72 (0.71, 4.16) | 0.231 |
| | (n=1,031) | (n=1,032) | | | | |
| | | | Unweighted MI | 0.7% (–0.6, 2.0) | 1.65 (0.68, 4.02) | 0.272 |
| | 1.2% | 0.9% | Weighted Complete cases | 0.4% (–0.5, 1.4) | 1.59 (0.11, 3.07) | 0.323 |
| | (11/913) | (7/817) | | | | |
* The p-value relates to the risk difference and these are coherent. The risk ratio is estimated from parameters of the same model but its confidence intervals are computed using a delta method transformation of the standard errors. As the proportions are close to 0, the transformation is highly nonlinear which gives a confidence interval for the risk ratio that is inconsistent with the p-value.
