# Supplementary material for: Internet-accessed sexually transmitted infection (e-STI) testing and results service: A randomised, single-blind, controlled trial
Source: PLoS Med. 2017 Dec 27;14(12):e1002479. doi: 10.1371/journal.pmed.1002479 (PMC5744909; doi:10.1371/journal.pmed.1002479)
Supplement: S3 Table — (PPTX) [file pmed.1002479.s009.pptx]

## Slide 1
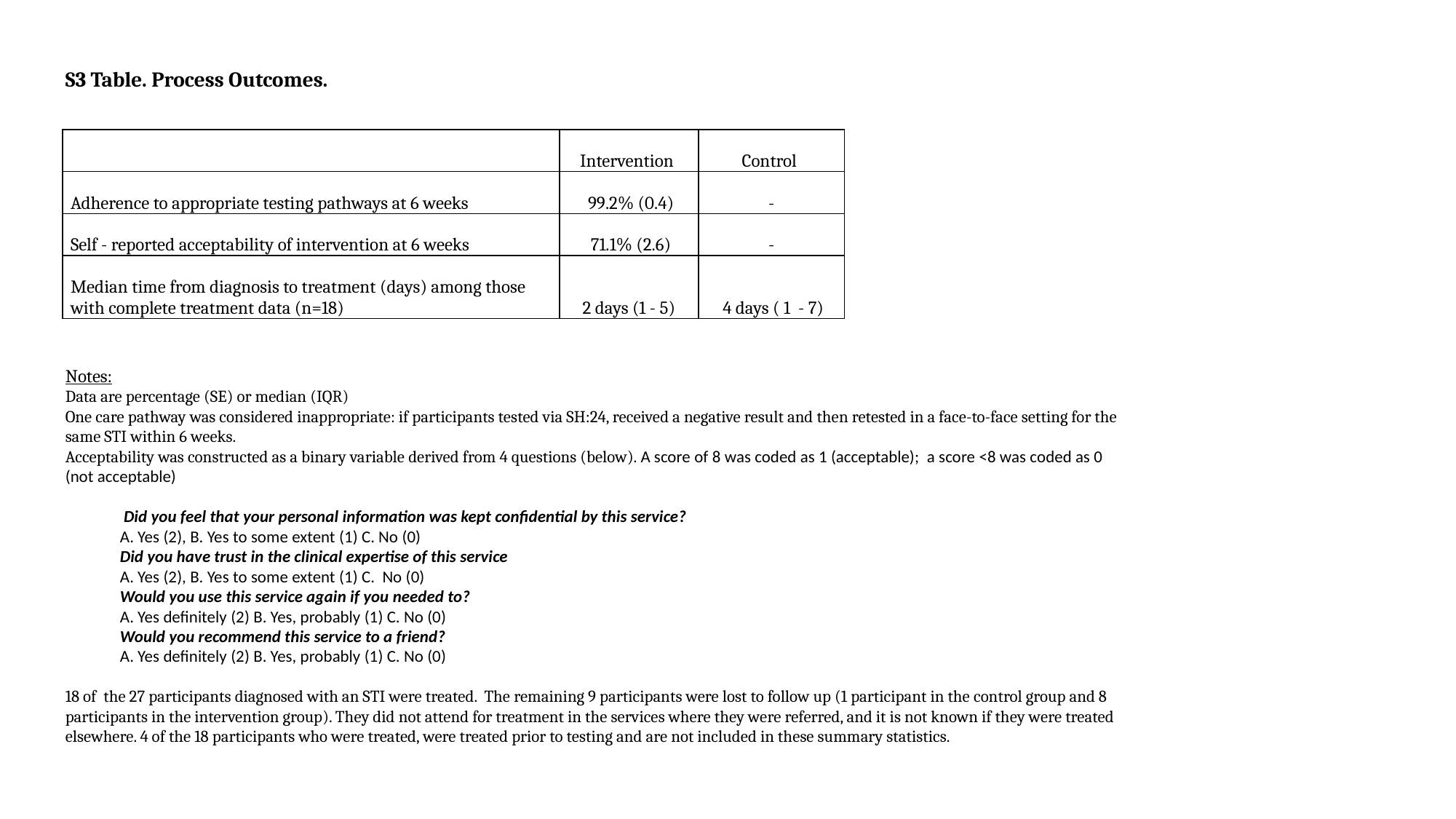

S3 Table. Process Outcomes.
| | Intervention | Control |
| --- | --- | --- |
| Adherence to appropriate testing pathways at 6 weeks | 99.2% (0.4) | - |
| Self - reported acceptability of intervention at 6 weeks | 71.1% (2.6) | - |
| Median time from diagnosis to treatment (days) among those with complete treatment data (n=18) | 2 days (1 - 5) | 4 days ( 1 - 7) |
Notes:
Data are percentage (SE) or median (IQR)
One care pathway was considered inappropriate: if participants tested via SH:24, received a negative result and then retested in a face-to-face setting for the same STI within 6 weeks.
Acceptability was constructed as a binary variable derived from 4 questions (below). A score of 8 was coded as 1 (acceptable); a score <8 was coded as 0 (not acceptable)
 Did you feel that your personal information was kept confidential by this service?
A. Yes (2), B. Yes to some extent (1) C. No (0)
Did you have trust in the clinical expertise of this service
A. Yes (2), B. Yes to some extent (1) C. No (0)
Would you use this service again if you needed to?
A. Yes definitely (2) B. Yes, probably (1) C. No (0)
Would you recommend this service to a friend?
A. Yes definitely (2) B. Yes, probably (1) C. No (0)
18 of the 27 participants diagnosed with an STI were treated. The remaining 9 participants were lost to follow up (1 participant in the control group and 8 participants in the intervention group). They did not attend for treatment in the services where they were referred, and it is not known if they were treated elsewhere. 4 of the 18 participants who were treated, were treated prior to testing and are not included in these summary statistics.
