# Supplementary material for: Internet-accessed sexually transmitted infection (e-STI) testing and results service: A randomised, single-blind, controlled trial
Source: PLoS Med. 2017 Dec 27;14(12):e1002479. doi: 10.1371/journal.pmed.1002479 (PMC5744909; doi:10.1371/journal.pmed.1002479)
Supplement: S1 Text — (PDF) [file pmed.1002479.s010.pdf]

Original Paper

Can Internet-Based Sexual Health Services Increase Diagnoses of Sexually Transmitted Infections (STI)? Protocol for a Randomized Evaluation of an Internet-Based STI Testing and Results Service

Emma Wilson[1], MSc; Caroline Free[1], MB ChB, MRCGP, PhD; Tim P Morris[2,3], PhD; Michael G Kenward[3], PhD; Jonathan Syred[4], MSc; Paula Baraitser[4], MD, FFSRH, FFPH

[1]Department of Population Health, Faculty of Epidemiology and Population Health, London School of Hygiene and Tropical Medicine, London, United Kingdom

[2]MRC Clinical Trials Unit at UCL, London, United Kingdom

[3]Department of Medical Statistics, Faculty of Epidemiology and Population Health, London School of Hygiene and Tropical Medicine, London, United Kingdom

[4]SH24 Evaluation Team, Kings Centre for Global Health, Kings College London, London, United Kingdom

Corresponding Author:
Emma Wilson, MSc
Department of Population Health
Faculty of Epidemiology and Population Health
London School of Hygiene and Tropical Medicine
Keppel Street
London, WC1E 7HT
United Kingdom
Phone: 44 207 299 4619
Fax: 44 207 436 5389
Email: emma.wilson@lshtm.ac.uk

Abstract

Background: Ensuring rapid access to high quality sexual health services is a key public health objective, both in the United Kingdom and internationally. Internet-based testing services for sexually transmitted infections (STIs) are considered to be a promising way to achieve this goal. This study will evaluate a nascent online STI testing and results service in South East London, delivered alongside standard face-to-face STI testing services.

Objective: The aim of this study is to establish whether an online testing and results services can (1) increase diagnoses of STIs and (2) increase uptake of STI testing, when delivered alongside standard face-to-face STI testing services.

Methods: This is a single-blind randomized controlled trial. We will recruit 3000 participants who meet the following eligibility criteria: 16-30 years of age, resident in the London boroughs of Lambeth and Southwark, having at least one sexual partner in the last 12 months, having access to the Internet and willing to take an STI test. People unable to provide informed consent and unable to read and understand English (the websites will be in English) will be excluded. Baseline data will be collected at enrolment. This includes participant contact details, demographic data (date of birth, gender, ethnicity, and sexual orientation), and sexual health behaviors (last STI test, service used at last STI test and number of sexual partners in the last 12 months). Once enrolled, participants will be randomly allocated either (1) to an online STI testing and results service (Sexual Health 24) offering postal self-administered STI kits for chlamydia, gonorrhoea, syphilis, and HIV; results via text message (short message service, SMS), except positive results for HIV, which will be delivered by phone; and direct referrals to local clinics for treatment or (2) to a conventional sexual health information website with signposting to local clinic-based sexual health services. Participants will be free to use any other interventions or services during the trial period. At 6 weeks from randomization we will collect self-reported follow-up data on service use, STI tests and results, treatment prescribed, and acceptability of STI testing services. We will also collect objective data from participating STI testing services on uptake of STI testing, STI diagnoses and treatment. We hypothesise that uptake of STI testing and STI diagnoses will be higher in the intervention arm. Our hypothesis is based on the assumption that the intervention is less time-consuming, more convenient, more private, and incur less stigma and embarrassment than face-to-face STI testing pathways. The primary outcome measure is diagnosis of any STI at 6 weeks from randomization

XSL•FO
RenderX

and our co-primary outcome is completion of any STI test at 6 weeks from randomization. We define completion of a test, as samples returned, processed, and results delivered to the intervention and/or clinic settings. We will use risk ratios to calculate the effect of the intervention on our primary outcomes with 95% confidence intervals. All analyses will be based on the intention-to-treat (ITT) principle.

Results: This study is funded by Guy's and St Thomas' Charity and it has received ethical approval from NRES Committee London-Camberwell St Giles (Ref 14/LO/1477). Research and Development approval has been obtained from Kings College Hospital NHS Foundation Trust and Guy's and St Thomas' NHS Foundation Trust. Results are expected in June 2016.

Conclusions: This study will provide evidence on the effectiveness of an online STI testing and results service in South East London. Our findings may also be generalizable to similar populations in the United Kingdom.

Trial Registration: International Standard Randomized Controlled Trial Number (ISRCTN): 13354298; http://www.isrctn.com/ISRCTN13354298 (Archived by WebCite at http://www.webcitation.org/6d9xT2bPj)

(JMIR Res Protoc 2016;5(1):e9) doi:10.2196/resprot.4094

KEYWORDS

sexually transmitted infections; eHealth; Internet; services

Introduction

Sexually transmitted infections (STIs) are an important cause of morbidity and mortality worldwide, and a key indicator of sexual ill health. Globally, incident cases of curable STIs (chlamydia, gonorrhoea, syphilis, and trichomonas vaginalis) rose from 448.3 million in 2005 to 498.9 million in 2008 [1]. While global incidence rates of HIV infection are on the decline, prevalence remains significant: UNAIDS estimated 2.3 million new HIV infections in 2012 and that 35.3 million people were living with HIV [2]. Moreover, HIV/AIDs represents 3.4% of the total global disease burden and is the seventh leading cause of all disability-adjusted life years worldwide [3].

In England, 448,422 new STI diagnoses were made in 2012, with higher rates recorded among young heterosexuals, men who have sex with men, and some black and ethnic minority groups [4]. Importantly, the patterning of STIs reflects stark health inequalities. A recent probability sample survey found that men and women from the most deprived areas of Britain had greater odds of testing positive for chlamydia (the most common STI) than those from wealthier areas (men, adjusted OR 3.42 (95%CI, 1.28-9.16), *P*=.003; women, adjusted OR 4.01(95%CI, 1.67-9.63), *P*=.008) [5].

Increasing diagnoses of untreated STIs is a key public health objective, both in the United Kingdom and internationally, to reduce onward transmission of infection and prevent long term health complications [6]. Increasing access to HIV testing and ensuring early diagnoses of HIV in high prevalent areas is of particular concern. In 2013, in the United Kingdom, it was estimated that a quarter of those living with HIV were unaware that they were infected and 42% of those diagnosed with HIV were diagnosed late, after the point at which treatment should have begun [7].

Internet-based STI testing is considered a promising means for increasing access to STI testing and reaching high-risk groups [8]. There are a number of models for Internet-based STI testing. For the purposes of this study we focus on a model which enables users to order a test kit online, take self-administered samples in their home, return test samples to a laboratory and receive their results via SMS text messaging (short message service, SMS), email or phone call. As far as we are aware, there have been no experimental studies to demonstrate the effectiveness of Internet-based testing services compared to standard face-to-face clinical pathways.

Observational studies have been encouraging, reporting high STI positivity among service users and reaching populations with a combination of both sociodemographic and behavioral risk factors. In the United States, Ladd and colleagues found that out of 205 rectal samples ordered and returned by women using the "iwantthekit" website between January 2009 and February 2011, 18.5% were positive for at least one STI [9]. The majority of women in the sample were single (91.2%), young (mean age 25.8 years) and of African-American ethnicity (50.0%). Half had never used condoms for rectal sex (48.7%). A study with male users of the same website found that of 501 STI kits returned by men over the age of 14, between September 2006 and May 2009, 21% tested positive for chlamydia, gonorrhoea or trichomonas vaginalis [10]. The majority of users were young (median age 24.5 years), single (84%), and either white (47%) or black ethnicity (45%). While these studies are promising, sample sizes have been small and no comparison has been made with users of face-to-face pathways.

In the United Kingdom there is limited evidence on Internet-based STI testing. One descriptive study reported trends in chlamydia testing among 15-24 year olds. It found that Internet tests, which have not been widely promoted, represented 5% of all tests within the National Chlamydia Screening Programme (NCSP) between 2006 and 2010 [11]. A higher proportion of Internet tests were positive compared to tests conducted in general practice services (7.6% vs 5.6%) but slightly lower than in community-based sexual and reproductive health services (7.6% vs 8.2%). Compared to testers in face-to-face settings, a higher proportion of Internet testers were men, of white ethnicity, and in the upper age group (20-24 years).

Recent exploratory qualitative studies with young people in the United Kingdom suggest that Internet or mobile applications of sexual health services are likely to be acceptable to this population, due to ease and convenience. Privacy, trust and credibility of websites or apps were highlighted as important

XSL•FO
RenderX

considerations for service development [12,13]. However, there is some uncertainty on whether Internet services can reach marginalized populations. In Scotland, Lorimer and McDaid [14] found that young men from more deprived areas seemed more disconnected from Internet technology and stated a preference for face-to-face services. This echoes findings from analysis of chlamydia testing data from England (discussed earlier), which found that a higher proportion of tests conducted in face-to-face services (GP and SRH clinics) were from more deprived areas, compared to Internet tests [11].

Given the limited evidence base it is difficult to draw conclusions on the effectiveness of Internet-based pathways in diagnosing a range of STIs compared to face-to-face service pathways.

Sexual Health 24 (SH:24) is an innovative Internet-based sexual health service that aims to improve access to sexual health services in the London boroughs of Southwark and Lambeth by addressing both supply and demand side barriers to care. Southwark and Lambeth have some of the highest rates of STIs in England, as well as high rates of teenage pregnancy and abortion [15]. Current face-to-face services are unable to meet demand with the result that many people are turned away from services.

In November 2014, SH:24 launched its first online product (minimal viable product 1) - an online STI testing and results service allowing users to order free postal STI kits, receive their results by SMS text messaging (or by phone in the event of a positive HIV result), and be referred on to specialist sexual health clinics for treatment. Over the course of this 4 year project, SH:24 will build in complexity, gradually adding increasing layers of functionality to the website, such as telephone support services and contraceptive services. SH:24 will be fully embedded within local sexual health economies ensuring that care pathways are integrated with clinical and other local services: for example by ensuring that users with acute STIs are signposted to face-to-face clinical care.

As an untested intervention, SH:24 has implications for the commissioning of sexual health services not only in London but also nationally. In line with national and international quality frameworks, SH:24 will be evaluated using a variety of data sources and methodologies to assess whether it delivers a safe, effective, patient-centered, timely, efficient, and equitable service [16,17]. This study will focus on establishing whether SH24 delivers an effective Internet-based STI testing and results service compared to face-to-face STI testing services, thus contributing to the international evidence base in this field.

Methods

Study Design

We will carry out a randomized controlled trial (Figure 1). Participants will be randomly allocated either (1) to a sexual health website (SH:24) offering free postal STI kits for chlamydia, gonorrhoea, syphilis and HIV, results via SMS text messaging (positive results for HIV will be delivered by phone), and direct referrals to clinic-based treatment options; or (2) to a conventional sexual health information website with signposting to local clinic-based sexual health services. Participants will be free to use any other service or intervention during the trial period.

Eligibility

Participants will be eligible if they are between 16 and 30 years of age, resident in the London boroughs of Lambeth and Southwark, sexually active (at least one sexual partner in the last 12 months), willing to take an STI test, and have access to the Internet (owner of a mobile phone or able to access a laptop, tablet, personal computer in their own home). Participants will be excluded if they are unable to read in English as the websites will be in English. Those unable to give informed consent, such as people with severe learning difficulties, will also be excluded.

Recruitment and Consent

This study is being conducted in community settings in the boroughs of Lambeth and Southwark in South East London. The study coordinator, together with a team of research assistants, will approach community networks, organizations, and institutions such as further education colleges, universities, patient groups, sexual health advocacy groups, sports centers, entertainment and leisure venues and major employers to recruit participants. We will also utilize social media sites popular among our study population. These will include Facebook, Twitter, and dating applications for gay men such as Scruff and Grindr.

After potential participants have been assessed for their eligibility, they will be provided with detailed verbal and written information about the study, and given the opportunity to ask any questions. If the participant agrees to participate, we will ask them to provide consent via the trial website (eg, using a mobile phone) or via paper-based forms. If potential participants would like more time to consider their involvement, we will give them the contact details of the study coordinator so that they can talk through any queries or doubts. Potential participants will also be able to access the study website independently, for example via social media sites.

XSL•FO
RenderX

Figure 1. Consolidated Standards of Reporting Trials (CONSORT) flow chart.

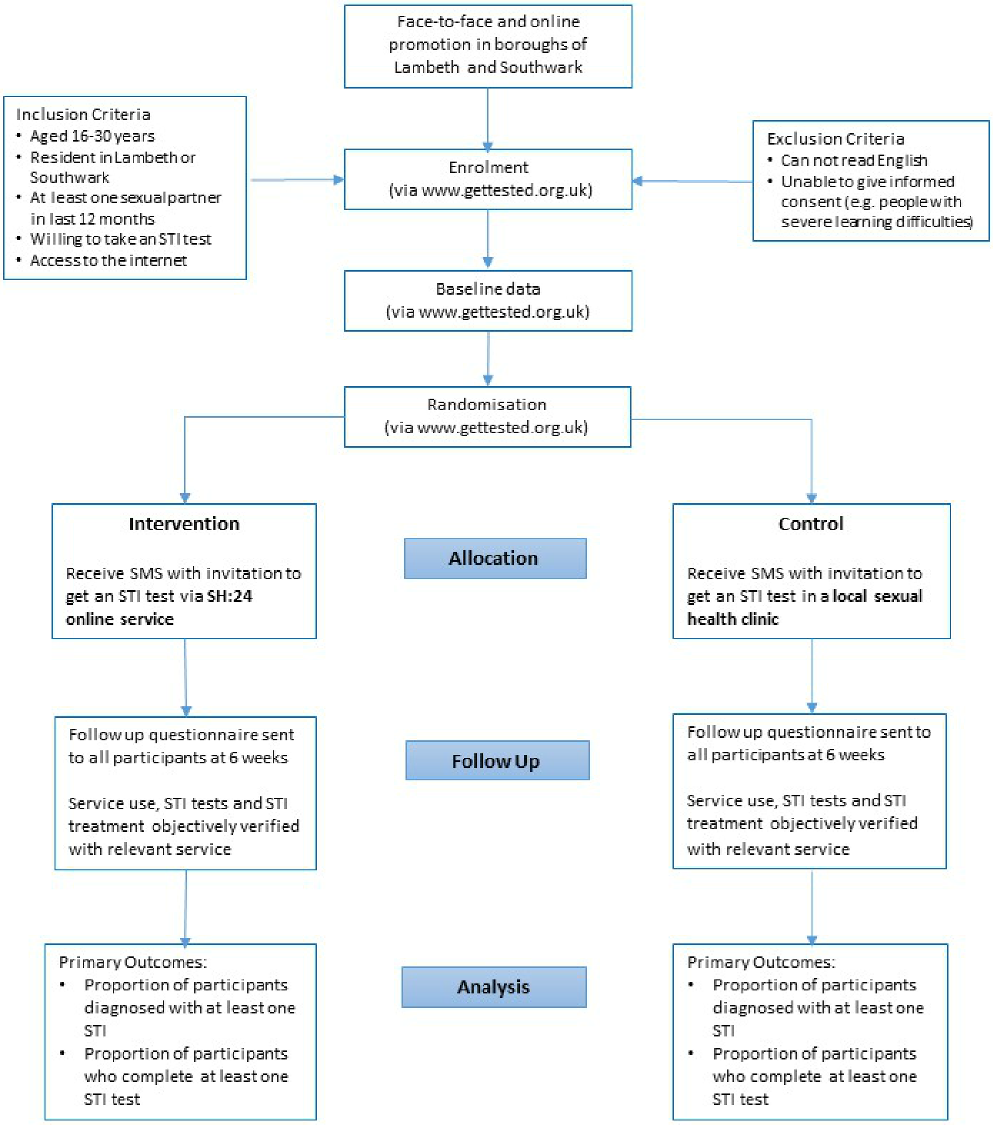

Allocation

Participants will be asked to enter baseline data directly onto the trial website or using paper-based forms. After baseline information has been submitted, an independent computer-based randomization program will generate a unique research number and allocate participants to either the intervention or control group. Participants will be sent a SMS text message with the URL of their allocation.

We will allocate by minimization, taking into account gender (male, female), age (16-19, 20-24, 25-30), number of sexual partners (1, 2+) and sexual orientation (men who have sex with men [MSM], all other groups), where all factors have equal weight in determining marginal imbalance. To introduce a random element, allocation will be weighted toward the underrepresented group using a probability of .8. In all other instances, participants will be allocated in a 1:1 ratio.

Due to the nature of the intervention, participants will know to which arm they have been allocated. Laboratory staff carrying out STI tests will process the tests as per routine care, and will be unaware that samples are linked to a randomized controlled trial. Researchers assessing the outcomes will be blind to the treatment allocation.

Intervention Group

Through a design-led and user-centered approach, SH:24 has created an appropriate Internet-based sexual health service with the aim of improving access to sexual health services in the boroughs of Southwark and Lambeth.

Participants in the intervention group will be directed to a website which will offer them: sexual health risk assessment; the opportunity to order self-administered sample collection kits for HIV, chlamydia, syphilis, gonorrhoea; results given by SMS text message and by phone (positive HIV result only); direct referral to local clinics for treatment; health promotion information; signposting to clinic and other social services.

Participants who test positive for chlamydia, syphilis or gonorrhoea infection will be sent a SMS text message with their result and details of local sexual health clinics where they will be able to obtain treatment. Participants who test positive for HIV will be informed by phone by a health professional.

XSL•FO
RenderX

Control Group

Participants in the control group will be directed to a sexual health information website with signposting to clinic-based services. These include the Camberwell Sexual Health Centre, the Burrell Street Sexual Health Centre, the Lloyd Clinic and a range of local community-based sexual and reproductive health services in Lambeth and Southwark, where they can obtain an STI test in person. The information website will provide the address, contact details, and location of the clinics (via a google map image), alongside the URL link to the clinic website.

All participants will be free to use any other services or interventions during the trial period.

Outcomes

Our primary outcomes will be the proportion of participants diagnosed with at least one STI in each arm at 6 weeks from randomization, and the proportion of participants who complete at least one STI test in each arm at 6 weeks from randomization. The secondary outcomes are listed in Textbox 1.

Textbox 1. Secondary outcomes.

- The proportion of STI tests that are positive in each arm
- The proportion of participants who are prescribed treatment in each arm
- Time from randomization to completion of an STI test in each arm
- Time from randomization to treatment in each arm
- Time from diagnosis to treatment in each arm
- The proportion of the intervention group who agree that Internet-based STI testing is acceptable
- The proportion of the intervention group who adhere to the prescribed Internet-based testing pathway, without seeking additional support from face-to-face services

Data Collection

Eligibility data will include age, postal code, independent access to the Internet (owner of mobile phone or access to tablet, laptop or computer at home), at least one sexual partner in the last 12 months, willingness to take an STI test.

Participants will be asked to enter their baseline data directly onto the trial website or using paper-based forms. We will collect the following: contact details including first name, surname, main mobile number, email address, and primary postal address; demographic data including date of birth, gender, ethnicity, and sexual orientation; sexual health behaviors including last STI test, service used at last STI test, and number of sexual partners in the last 12 months

Self-reported follow up data at 6 weeks will be collected by post or online, according to the preference of participants. We will assess whether Internet-based testing was acceptable to participants and collect data on service use, STI tests and results, and whether treatment was prescribed. We will also collect objective data on STI tests, results, and treatment from SH:24 and local sexual health services, with participants' prior consent.

Lab Processing

The Doctors Laboratory (TDL) will process all returned samples for the intervention group and the results will be captured by SH:24 data systems. Samples taken in face-to-face settings will be processed as per routine care. The study coordinator will contact the local services and SH:24 to obtain participants' test results and treatment details, with prior consent. These data will be anonymized and uploaded into the secure trial website.

Follow-Up

We will utilize evidence-based methods identified in systematic reviews to minimize losses to follow up [18]. These include providing incentives to all participants, contacting respondents prior to sending questionnaires and contacting nonresponders using phone call, texting, email, and post. The research team will verify participant addresses at enrolment or shortly after, and attempt to contact participants who have provided an incomplete or unknown address.

To maximize participation and response rates we will provide an incentive (such as a lollipop or chocolate) at enrolment and an unconditional incentive of £5 at 6 weeks when we send out the follow-up questionnaire. Participants will then receive a further £5, on receipt of their completed questionnaires. They will be informed of the incentives at enrolment. Our incentives will not exceed a monetary value of £15 per participant.

Sample Size

The study is powered for the first of our primary outcome measures which is the proportion of participants diagnosed with at least one STI in each arm. Two factors determine the number of participants needed for this trial: the estimated proportion of participants with an STI and the size of the treatment effect.

Our estimates are based on the following data:

The Greenwich sexual health service has demonstrated a 50% return rate among users who order test kits online (personal communication Dr David Pinson, Health Improvement Principal, Royal Borough of Greenwich). Eligibility for this study is restricted to people who are willing to take an STI test. However, not all of those allocated to the intervention group are likely to order a test kit. We estimate that 30% will not complete this first step. Among the 70% who order a kit, we assume that 50% will return the kit for analysis, based on the Greenwich data. Following these assumptions, 35% of the intervention group are likely to complete an STI test.

There are no available data which would give us an estimate of the likely numbers that will get tested in the control group.

XSL•FO
RenderX

However, we assume that far fewer people (10%) are likely to seek a test in clinic-based settings.

Chlamydia is the most commonly diagnosed STI of the four STIs of interest in this study both at the national level (England) and at the local authority level (Lambeth and Southwark) [4,15]. We based our prevalence estimates on the proportion of positive chlamydia tests among 15-24 year olds in general practice settings in Lambeth and Southwark, which was 6% in 2012 [19].

We based our estimated losses to follow up on previous eHealth studies in the United Kingdom which have achieved 90% follow up [20].

A sample size of 3000 would have 90% power (two-sided alpha=5%) to detect a relative risk of 3.5, (2.1% risk of diagnosis in the intervention group vs 0.6% risk of diagnosis in the control group), allowing for 10% losses to follow up. This equates to 10% of the control group being tested, with a 6% probability of infection as in general practice settings and 35% of the intervention tested with a 6% probability of infection as in general practice settings.

With regard to our co-primary outcome measure, with 3000 participants we would have 99% power (two-sided alpha=5%) to detect an absolute difference of 25% between the proportion of participants who complete a test in the intervention group versus the proportion who complete a test in the control group (35% versus 10%).

Statistical Methods

The analysis of data will adhere to the prespecified statistical analysis plan outlined below. The analyses of the co-primary outcomes are described in detail; analyses for other outcomes follow the same principles unless otherwise specified.

There are no planned interim analyses and so no rules for stopping early. Analyses comparing the interventions will follow the ITT principle as far as possible [21]. Analyses will include participants with no missing outcome data in their randomized groups. Any estimate described below will be accompanied by 95% confidence intervals.

The primary analysis of the first co-primary outcome (STI diagnosis) will estimate the proportion of STI diagnoses for the SH:24 vs conventional sexual health services via a risk ratio. Treatment allocation balances gender, age, number of sexual partners in the last 12 months, and sexual orientation, and these need to be reflected in the analysis. This will be done by weighting on the inverse propensity score (estimated by logistic regression) to reduce the variance of estimates and obtain confidence intervals of the correct width [22].

Some outcome data are expected to be missing. Missing data may occur if participants do not complete a 6-week follow-up questionnaire and attend a different sexual health service (ie, not a local clinic or SH:24) . The principle analysis will assume that the distribution of STI diagnoses among these participants is identical to those with observed data, conditional on propensity scores–missing at random–and so will be based on the weighted analysis of the complete cases. Sensitivity analyses assuming departures from missing at random will proceed via multiple imputation of outcome, using inverse probability weighting on the estimated propensity score and with allocated group as the only covariate. Assuming “missing not at random” mechanisms, the odds of STI diagnoses for missing participants will be varied to be ¼, ½, 2 and then 4 times larger than in the missing at random (MAR) analysis. This will be done in a factorial manner, separately for each arm. We judge these fractions to be reasonable, though a value of ¼ in one arm and 4 in the other (or vice versa) is at the boundary of what is plausible. The risk difference (rather than ratio), weighted by the inverse propensity score, and the proportion of STI diagnoses in each arm will also be presented. Finally, the proportion of STI tests taken that are positive will be summarized by arm, though no comparison of the groups will be given because this analysis excludes individuals based on a variable that will be heavily influenced by randomization.

The primary analysis of the second co-primary outcome (completion of an STI test) and secondary outcome (prescribed treatment) will follow the same principles as above. Again, the risk ratio, risk difference, and proportions in each arm will be reported.

The time from randomization to (1) test completion and (2) treatment are of interest. Therefore, for each measure we will estimate the restricted-mean survival time (RMST) in each arm, setting the restricted mean time t^*=6 weeks for time to test and t^*=3 months for time to treatment. This will be estimated from a “3df/1df” Royston-Parmar model and the difference in restricted-mean survival time estimated [23]. The median survival time from diagnosis to treatment in each arm will also be summarized. No comparison will be made between groups as this analysis excludes individuals based on a variable (STI diagnosis) that is heavily influenced by randomization.

For the SH:24 group, the proportion of participants who deem Internet-based testing to be acceptable will be summarized, as will the proportion who adhere to the SH:24 testing pathway.

It is possible that differences between groups will vary according to age, level of deprivation, sexuality, ethnicity, and gender. Subgroup analyses will be done for these characteristics and interaction tests will be performed. These will have low impact as we anticipate any interactions will be small. Further, these analyses will be regarded strictly as hypothesis-generating.

Ethical Arrangements

Informed Consent

All participants recruited into the trial will be provided with information about the study (online or in hard copy) and given the opportunity to ask questions and clarify queries at the time of recruitment and subsequently with the study coordinator by email or by phone. The recruiting staff will check that participants are aware that consenting to participate means that they will be encouraged to undertake an STI test.

Participants’ Rights

Participants will be able to contact the trial co-ordinating center at Kings College London by email or by phone with any queries or doubts for the duration of the trial. If they request to be withdrawn from the study their status will be changed to

XSL•FO
RenderX

“withdrawn” on the study website and they will be excluded from participant lists for follow up.

Personal details will be stored on a password protected computer held on a secure server at Kings College London. This information will be stored separately from any anonymized research data, and will be deleted at the end of the study.

Participants in the intervention arm who report symptoms of STIs will be advised to see a health professional in a clinic-based setting. If they wish to continue with the Internet-testing pathway they will be allowed to do so. Participants in the intervention arm will be informed about any positive results for chlamydia, gonorrhoea, and syphilis by SMS text message and they will be texted information of local clinics where they can receive treatment. Participants who test positive for HIV will be informed by telephone by a trained health professional and will be referred on to specialist HIV services.

Participants' Safety

The intervention provides an opportunity to obtain a postal STI kit, notification of STI diagnoses, and opportunities for STI treatment. It is unlikely to cause any harmful effects. Participants who lack privacy in their home or participants who are in abusive interpersonal relationships may risk possible consequences if they participate in Internet-based STI testing. However, this risk will be minimized as we will ensure that at recruitment participants have sufficient privacy to participate in the trial. Furthermore, the website can be accessed via devices such as mobile phones and postal test kits will be sent out in packages that do not have any identifying features. Other large scale studies using Internet-based testing have not reported any related safety concerns [24]. The intervention website will also provide clear signposting to counselling services for violence as well as contraception and abortion services.

All sexual health services participating in this trial routinely deliver test results via SMS text message. There is a small risk that friends and partners may see participants’ results if phones are shared. However, as in routine care, if participants are concerned about their privacy they can opt to receive their results via different methods (eg, via post). The intervention delivers the results via SMS text message (except in the case of a positive result for HIV, which is delivered via phone). However, this is made very clear on the website and participants can choose not to order an STI test kit online, if they are concerned about their privacy.

Retention of Trial Documentation

We will retain the trial documentation for 10 years.

Results

By April 21, 2015, 1405 participants were randomized. We are currently recruiting and a timeline of our trial is included in Table 1.

Table 1. Trial timeline.

Timeline	Tasks
Months 1-6 (April-September 2014)	Trial setup
By month 6 (September, 2014)	Intervention website user tested and finalized (to be developed and finalized by SH:24)<br>Completed and user tested trial database and randomization system<br>Recruitment strategy designed and completed
By month 16 (July, 2015)	Recruitment to the trial completed
By month 22 (January 2016)	All follow up completed
By month 24 (March, 2016)	Data cleaned, trial database closed
By month 27 (June, 2016)	Analysis of trial results completed<br>Paper submitted for publication

Discussion

This trial will provide a robust and rigorous evaluation of a nascent online STI testing service in an area of South East London, characterized by poor levels of sexual health relative to the rest of the country. It will assess the added contribution of this service with respect to two distinct but interrelated outcomes: diagnoses of STIs and uptake of STI testing.

We envision that our findings will be highly policy-relevant and will be well-placed to inform decision-making for the effective commissioning and delivery of STI testing services in London. Our findings may also be generalizable to similar populations in the United Kingdom.

To our knowledge, this is the first RCT of an Internet-based testing service, which makes a direct comparison with standard face-to-face care. The findings from this study will therefore make a timely contribution to the international evidence base in this field.

XSL•FO
RenderX

Acknowledgments

We would like to thank the following individuals for their helpful comments on earlier drafts of this manuscript: Richard Hayes (Professor of Epidemiology and International Health, LSHTM), Dr Andrew Copas (Reader in Statistics, UCL), Dr David Prieto (Lecturer, LSHTM), Dr Catherine Lowndes (Consultant Scientist, PHE), Sarah Woodhall (Senior Surveillance Scientist, PHE) and Dr Salma Ayis (Lecturer in Medical Statistics, KCL).

Conflicts of Interest

Dr Baraitser is a director of SH:24 which is the community interest company that holds the grant from Guy's and St Thomas' Charity to develop the intervention. Additional oversight for the evaluation is provided by the Evaluation Steering Group and an independent adviser, Dr Caroline Free, based at the London School of Hygiene and Tropical Medicine.

References

1. Rowley J, Toskin I, Ndowa F. Global Prevalence and Incidence of Selected Curable Sexually Transmitted Infections - 2008. Geneva: WHO; 2012. URL: http://apps.who.int/iris/bitstream/10665/75181/1/9789241503839_eng.pdf?ua=1 [accessed 2015-07-01] [WebCite Cache ID 6ZgDxeI1N]
2. Joint United Nations Programme on HIV/AIDS (UNAIDS). Global report: UNAIDS Report on the Global AIDS Epidemic 2013 URL: http://www.unaids.org/sites/default/files/media_asset/UNAIDS_Global_Report_2013_en_1.pdf [accessed 2015-07-01] [WebCite Cache ID 6ZgE4hOsA]
3. World Health Organization. June. Geneva: WHO; 2014. Global Health Estimates Summary Tables: DALYs by Cause, Age and Sex, by WHO region, 2000-2012 URL: http://www.who.int/healthinfo/global_burden_disease/estimates/en/index2.html [accessed 2015-06-30] [WebCite Cache ID 6ZgEENpOi]
4. Public Health England. Sexually Transmitted Infections and Chlamydia Screening in England, 2012 HPR 7(23), 7 June 2013 HIV-STIs. England; 2013. Health Protection Report, weekly report URL: http://webarchive.nationalarchives.gov.uk/20140714084352/http://www.hpa.org.uk/hpr/archives/2013/hpr2313.pdf [accessed 2015-06-30] [WebCite Cache ID 6ZgETgTtM]
5. Sonnenberg P, Clifton S, Beddows S, Field N, Soldan K, Tanton C, da Silva Filomeno Coelho, et al. Prevalence, risk factors, and uptake of interventions for sexually transmitted infections in Britain: findings from the National Surveys of Sexual Attitudes and Lifestyles (Natsal). Lancet 2013 Nov 30;382(9907):1795-1806 [FREE Full text] [doi: 10.1016/S0140-6736(13)61947-9] [Medline: 24286785]
6. Department of Health and cross Government. A Framework for Sexual Health Improvement in England. London: Department of Health; 2013. URL: https://www.gov.uk/government/uploads/system/uploads/attachment_data/file/142592/9287-2900714-TSO-SexualHealthPolicyNW_ACCESSIBLE.pdf [accessed 2015-06-30] [WebCite Cache ID 6ZgEin59w]
7. Yin Z, Brown AE, Hughes G, Nardone A, Gill ON, Depeche VC. HIV in the United Kingdom: 2014 Report. England, London: Public Health England; 2014. URL: https://www.gov.uk/government/uploads/system/uploads/attachment_data/file/401662/2014_PHE_HIV_annual_report_draft_Final_07-01-2015.pdf [accessed 2015-06-30] [WebCite Cache ID 6ZgEtEToY]
8. European Centre for Disease Prevention and Control. Novel approaches to testing for sexually transmitted infections, including HIV and hepatitis B and C in Europe. Stockholm: ECDC; 2012. URL: http://ecdc.europa.eu/en/publications/Publications/Novel_approaches_to_testing_for_STIs_.pdf [accessed 2015-04-29] [WebCite Cache ID 6Y9XUCuFl]
9. Ladd J, Hsieh Y, Barnes M, Quinn N, Jett-Goheen M, Gaydos CA. Female users of internet-based screening for rectal STIs: descriptive statistics and correlates of positivity. Sex Transm Infect 2014 Sep;90(6):485-490 [FREE Full text] [doi: 10.1136/sextrans-2013-051167] [Medline: 24604333]
10. Chai SJ, Aumakhan B, Barnes M, Jett-Goheen M, Quinn N, Agreda P, et al. Internet-based screening for sexually transmitted infections to reach nonclinic populations in the community: risk factors for infection in men. Sex Transm Dis 2010 Dec;37(12):756-763 [FREE Full text] [doi: 10.1097/OLQ.0b013e3181e3d771] [Medline: 20644498]
11. Woodhall SC, Sile B, Talebi A, Nardone A, Baraitser P. Internet testing for Chlamydia trachomatis in England, 2006 to 2010. BMC Public Health 2012;12:1095 [FREE Full text] [doi: 10.1186/1471-2458-12-1095] [Medline: 23253518]
12. Gkatzidou V, Hone K, Gibbs J, Sutcliffe L, Sadiq ST, Sonnenberg P, et al. A user-centred approach to inform the design of a mobile application for STI diagnosis and management. In: Proceedings of the 27th International BCS Human Computer Interaction Conference (HCI 2013). 2013 Sep Presented at: The 27th International BCS Human Computer Interaction Conference (HCI 2013); 9 - 13 September 2013; London p. 1-6 URL: http://ewic.bcs.org/upload/pdf/ewic_hci13_short_paper8.pdf [WebCite Cache]
13. Fuller SS, Aicken C, Sutcliffe LJ, Estcourt CS, Gkatzidou V, Hone K, et al. O22.4 What Are Young People's Perceptions of Using Electronic Self-Tests For STIs Linked to Mobile Technology For Diagnosis and Care (eSTI2)? Sexually Transmitted Infections 2013 Jul 13;89(Suppl 1):A69-A70. [doi: 10.1136/sextrans-2013-051184.0212]
14. Lorimer K, McDaid L. Young men's views toward the barriers and facilitators of Internet-based Chlamydia trachomatis screening: qualitative study. J Med Internet Res 2013;15(12):e265 [FREE Full text] [doi: 10.2196/jmir.2628] [Medline: 24300158]

XSL•FO
RenderX

15. Public Health England. Sexual and Reproductive Health Profiles. URL: http://fingertips.phe.org.uk/profile/sexualhealth/data [accessed 2015-04-29] [WebCite Cache ID 6Y9XkQ1U0]
16. British Association for Sexual Health and HIV (BASHH). Standards for the Management of Sexually Transmitted Infections (STIs) - January 2010. London: MedFASH; 2010. URL: http://www.bashh.org/documents/2513.pdf [accessed 2015-04-29] [WebCite Cache ID 6Y9Y2TfKd]
17. Committee on Quality of Health Care in America, Institute of Medicine. Crossing the quality chasm: a new health system for the 21st century. Washington, D.C: National Academy Press; 2001.
18. Edwards P, Roberts I, Clarke M, DiGuiseppi C, Pratap S, Wentz R, et al. Increasing response rates to postal questionnaires: systematic review. BMJ 2002 May 18;324(7347):1183 [FREE Full text] [Medline: 12016181]
19. Public Health England. National Chlamydia Screening Programme. Chlamydia testing data for 15-24 year olds in England. January to December 2012 URL: http://www.chlamydiascreening.nhs.uk/ps/data.asp [accessed 2015-04-29] [WebCite Cache ID 6Y9vJ8fjd]
20. Free C, Knight R, Robertson S, Whittaker R, Edwards P, Zhou W, et al. Smoking cessation support delivered via mobile phone text messaging (txt2stop): a single-blind, randomised trial. Lancet 2011 Jul 2;378(9785):49-55 [FREE Full text] [doi: 10.1016/S0140-6736(11)60701-0] [Medline: 21722952]
21. White IR, Carpenter J, Horton NJ. Including all individuals is not enough: lessons for intention-to-treat analysis. Clin Trials 2012 Aug;9(4):396-407 [FREE Full text] [doi: 10.1177/1740774512450098] [Medline: 22752633]
22. Williamson EJ, Forbes A, White IR. Variance reduction in randomised trials by inverse probability weighting using the propensity score. Stat Med 2014 Feb 28;33(5):721-737 [FREE Full text] [doi: 10.1002/sim.5991] [Medline: 24114884]
23. Royston P, Parmar Mahesh K B. The use of restricted mean survival time to estimate the treatment effect in randomized clinical trials when the proportional hazards assumption is in doubt. Stat Med 2011 Aug 30;30(19):2409-2421. [doi: 10.1002/sim.4274] [Medline: 21611958]
24. Greenland KE, Op de Coul Eline L M, van Bergen Jan E A M, Brouwers Elfi E H G, Fennema Han J S A, Götz HM, Hoebe Christian J P A, van Ravesteijn Sander M, van den Broek Ingrid V F. Acceptability of the internet-based Chlamydia screening implementation in the Netherlands and insights into nonresponse. Sex Transm Dis 2011 Jun;38(6):467-474. [doi: 10.1097/OLQ.0b013e318204546e] [Medline: 21217416]

Abbreviations

HIV: human immunodeficiency virus
ITT: intention-to-treat
MAR: missing at random
MSM: men who have sex with men
NCSP: National Chlamydia Screening Programme
OR: odds ratio
RMST: restricted-mean survival time
SMS: short message service
STI: sexually transmitted infection
TDL: The Doctors Laboratory

Edited by G Eysenbach; submitted 24.12.14; peer-reviewed by J Bailey; comments to author 21.02.15; revised version received 30.04.15; accepted 30.04.15; published 15.01.16

Please cite as:
Wilson E, Free C, Morris TP, Kenward MG, Syred J, Baraitser P
Can Internet-Based Sexual Health Services Increase Diagnoses of Sexually Transmitted Infections (STI)? Protocol for a Randomized Evaluation of an Internet-Based STI Testing and Results Service
JMIR Res Protoc 2016;5(1):e9
URL: http://www.researchprotocols.org/2016/1/e9/
doi:10.2196/resprot.4094
PMID:26772143

©Emma Wilson, Caroline Free, Tim P Morris, Michael G Kenward, Jonathan Syred, Paula Baraitser. Originally published in JMIR Research Protocols (http://www.researchprotocols.org), 15.01.2016. This is an open-access article distributed under the terms of the Creative Commons Attribution License (http://creativecommons.org/licenses/by/2.0/), which permits unrestricted use, distribution, and reproduction in any medium, provided the original work, first published in JMIR Research Protocols, is

XSL•FO
RenderX

properly cited. The complete bibliographic information, a link to the original publication on http://www.researchprotocols.org, as well as this copyright and license information must be included.

XSL•FO
RenderX
