# Supplementary material for: Internet-accessed sexually transmitted infection (e-STI) testing and results service: A randomised, single-blind, controlled trial
Source: PLoS Med. 2017 Dec 27;14(12):e1002479. doi: 10.1371/journal.pmed.1002479 (PMC5744909; doi:10.1371/journal.pmed.1002479)
Supplement: S2 Text — (PDF) [file pmed.1002479.s011.pdf]

Corrigenda and Addenda

Addendum to: Can Internet-Based Sexual Health Services Increase Diagnoses of Sexually Transmitted Infections (STI)? Protocol for a Randomized Evaluation of an Internet-Based STI Testing and Results Service

Emma Wilson[1], MSc; Caroline Free[1], MB ChB, MRCGP, PhD; Tim P Morris[2,3], PhD; Jonathan Syred[4], MSc; Paula Baraitser[4], MD, FFSRH, FFPH

[1]Department of Population Health, Faculty of Epidemiology and Population Health, London School of Hygiene and Tropical Medicine, London, United Kingdom

[2]MRC Clinical Trials Unit at UCL, London, United Kingdom

[3]Department of Medical Statistics, Faculty of Epidemiology and Population Health, London School of Hygiene and Tropical Medicine, London, United Kingdom

[4]SH24 Evaluation Team, Kings Centre for Global Health, Kings College London, London, United Kingdom

Corresponding Author:
Emma Wilson, MSc
Department of Population Health
Faculty of Epidemiology and Population Health
London School of Hygiene and Tropical Medicine
Keppel Street
London, WC1E 7HT
United Kingdom
Phone: 44 0207 299 4619
Fax: 44 0207 436 5389
Email: emma.wilson@lshtm.ac.uk

Related Article:

Correction of: http://www.researchprotocols.org/2016/1/e9/

(JMIR Res Protoc 2016;5(3):e141) doi:10.2196/resprot.6280

Following the advice of the Trial Steering Committee, the authors of "Can Internet-Based Sexual Health Services Increase Diagnoses of Sexually Transmitted Infections (STI)? Protocol for a Randomized Evaluation of an Internet-Based STI Testing and Results Service" (JMIR Res Protoc 2016;5[1]:e9) are adding additional information about their primary and secondary analyses, as well as their respective outcomes, to provide readers with more details on the study.

Primary Analysis

For the primary analysis we will use multivariate imputation using chained equations (MICE) which uses the observed predictors of outcome and the predictors of loss to follow up to impute missing outcome data, thus attempting to correct for any potential bias caused by missing data under the assumption that data are ‘missing at random’.

Missing data will occur if:

1. Participants do not complete a 6-week follow up questionnaire (or submit an incomplete questionnaire), and attend a different health service (ie, not a clinic in Lambeth and Southwark or SH:24).
2. Participants who report testing for an STI but whose patient records we are unable to access (because they did not test in a clinic in Lambeth or Southwark or SH24 and they did not tell us where they tested so we were unable to obtain data from the clinic where they were tested).
3. Participants who are diagnosed with an STI but there is no record of them attending any clinic in Lambeth and Southwark for treatment and they did not tell us where they obtained treatment so we were unable to obtain data regarding whether or not they were treated.

We will impute each of the incomplete outcome variables using multivariate imputation using chained equations. Sexuality is also incomplete but is a baseline variable, so a missing category will be used. The propensity score for randomised allocation will be estimated for all participants using a logistic regression model with randomised group as the response, and gender, age

XSL•FO
RenderX

(years), number of sexual partners in the last 12 months, sexual orientation and ethnicity as covariates. The imputation model will then contain randomised group as a covariate and will be weighted by the inverse of the estimated propensity score. The imputation model for any incomplete variable will then condition on other incomplete variables. In particular, the conditional model to impute testing according to clinic data will include self-reported testing; and the model to impute treated STI will include diagnosis of STI. One hundred imputed datasets will be generated. Multiple imputation inference will then proceed via Rubin's rules [1].

We will account for baseline factors (gender, age, number of sexual partners in last 12 months, sexuality and ethnicity) by weighting on the inverse propensity score, which we will estimate by logistic regression. This will allow us to obtain more precise estimates and confidence intervals with the correct coverage.

Secondary Analyses

Sensitivity to Missing Outcome Data

We will perform a sensitivity analysis to explore departures from MAR assumptions. We will multiply impute missing outcome data, using inverse probability weighting on the estimated propensity score and with allocated group and whether or not participants report having been tested as covariates. The odds of STI diagnosis and the odds of a completed STI test for missing participants will be varied to be ¼, ½, 2 and then 4 times larger than the MAR analyses.

The risk difference and risk ratios weighted by inverse propensity score will be reported alongside proportions.

Subgroup Analyses

In order to explore heterogeneity of the intervention effect on our primary outcomes, we will test for interaction at a 5% level of significance to assess whether effectiveness varies by:

- Gender
  - Male
  - Female
- Ethnicity
  - White
  - Asian/Asian British
  - Black/African/Caribbean/Black British
  - All other groups (Mixed/Multiple ethnic groups/Other ethnic group)
- Sexuality
  - Men who have sex with men
  - All other groups
- Age group
  - 16-19 years
  - 20-24 years
  - 25-30 years
- Control group
  - Time period when SH:24 website available exclusively to intervention group participants
  - Time period when SH:24 website unblocked

We will test for linear interaction for deprivation (centiles of overall UK Indices of Multiple Deprivation ranks) using a log binomial model. These analyses will be conducted in the complete cases under a missing-at-random assumption. As with the primary analyses, they will be weighted by the inverse of the estimated propensity score. When a subgroup variable is one that appears in the propensity score (defined above), we will re-estimate the propensity score omitting the subgroup variable.

Intervention effect estimates by subgroups will be presented in a forest-type plot. Given that the study is not powered to test for interactions, these analyses will be treated as exploratory and the statistical significance of the interactions will be interpreted with caution.

Secondary Outcomes

The primary analysis of the following secondary outcome will follow the same principles as the analysis of our co-primary outcomes described above:

- The proportion of participants who are prescribed treatment in each arm

For our time-to-event secondary outcomes we will conduct the following analyses:

We will use survival analysis to estimate time from randomisation to (1) test completion and (2) treatment. For each measure we will estimate the restricted mean survival time (RMST) setting the restricted mean time t*=6 weeks (42 days) for time to test and t*= 3 months (84 days) for time to treatment.

This will be estimated from a "3df/1df" Royston–Parmar model and the difference in restricted mean survival time will be estimated.

Process Outcomes

For the following process outcomes we will summarise:

- The proportion of STI tests taken that are positive in each arm
- The median survival time from diagnosis to treatment in each arm
- The proportion of the intervention group who deem the intervention to be acceptable
- The proportion of the intervention group who adhere to an appropriate testing pathway
- The proportion of participants who complete an STI test, by service type
- The proportion of participants who are diagnosed with an STI, by service type

These estimates will be summarised by arm but there will be no comparison of groups.

Acceptability will be constructed as a binary variable, derived from 4 questions. A score of 8 will be coded as 1 (acceptable); a score <8 will be coded as 0 (not acceptable).

Did you feel that your personal information was kept confidential by this service?

XSL•FO
RenderX

A. Yes (2), B. Yes to some extent (1) C. No (0)

Did you have trust in the clinical expertise of this service

A. Yes (2), B. Yes to some extent (1) C. No (0)

Would you use this service again if you needed to?

A. Yes definitely (2) B. Yes, probably (1) C. No (0)

Would you recommend this service to a friend?

A. Yes definitely (2) B. Yes, probably (1) C. No (0)

Reference

1. Rubin DB. Multiple Imputation for Nonresponse in Surveys. New York: Wiley & Sons 1987.

Edited by G Eysenbach; This is a non-peer-reviewed article.submitted 28.06.16; accepted 28.06.16; published 10. 08.16

Please cite as:
Wilson E, Free C, Morris TP, Syred J, Baraitser P
Addendum to: Can Internet-Based Sexual Health Services Increase Diagnoses of Sexually Transmitted Infections (STI)? Protocol for a Randomized Evaluation of an Internet-Based STI Testing and Results Service
JMIR Res Protoc 2016;5(3):e141
URL: http://www.researchprotocols.org/2016/3/e141/
doi:10.2196/resprot.6280
PMID:27783558

©Emma Wilson, Caroline Free, Tim P Morris, Jonathan Syred, Paula Baraitser. Originally published in JMIR Research Protocols (http://www.researchprotocols.org), 10.08.2016. This is an open-access article distributed under the terms of the Creative Commons Attribution License (http://creativecommons.org/licenses/by/2.0/), which permits unrestricted use, distribution, and reproduction in any medium, provided the original work, first published in JMIR Research Protocols, is properly cited. The complete bibliographic information, a link to the original publication on http://www.researchprotocols.org, as well as this copyright and license information must be included.

XSL•FO
RenderX
